# Determination of the spatial susceptibility to Yellow Fever using a multicriteria analysis

**DOI:** 10.1590/0074-02760180509

**Published:** 2019-05-06

**Authors:** Camilla Adriane de Paiva, Adriana Paulo de Sousa Oliveira, Samuel Soares Muniz, Maria Lúcia Calijuri, Vitor Juste dos Santos, Sabrina do Carmo Alves

**Affiliations:** 1 Universidade Federal de Ouro Preto, Programa de Pós-Graduação em Engenharia Ambiental, Ouro Preto, MG, Brasil; 2 Universidade Federal de Viçosa, Programa de Pós-Graduação em Engenheira Civil, Viçosa, MG, Brasil; 3 Universidade Federal de Viçosa, Departamento de Engenharia Civil, Viçosa, MG, Brasil

**Keywords:** geographic information systems, risk estimation, vulnerability map, epidemiology

## Abstract

**BACKGROUND:**

The outbreak of sylvatic Yellow Fever (SYF) in humans during 2016-2017 in Brazil is one of the greatest in the history of the disease. The occurrence of the disease in areas with low vaccination coverage favoured the dissemination of the disease; therefore, it is necessary to identify the areas vulnerability to the YF virus (YFV) to assist in the adoption of preventive measures.

**OBJECTIVE:**

To correlate the physical-environmental elements associated with the occurrence of SYF in humans via a multicriteria analysis.

**METHODS:**

For the multicriteria analysis, preponderant elements related to SYF occurrences, including soil usage and coverage, temperature, precipitation, altitude, mosquito transmitters, and non-human primate occurrence areas, were considered. The results were validated by assessing the correlation between the incidence of SYF and the vulnerable areas identified in the multicriteria analysis.

**RESULTS:**

Two regions with different vulnerability to the occurrence of the disease were identified in the multicriteria analysis, with emphasis on the southern areas of the state of São Paulo northeast areas of Minas Gerais, and the entire states of Rio de Janeiro and Espírito Santo. The map of SYF vulnerability obtained in the multicriteria analysis coincides with the areas in which cases of the disease have been recorded. The regions that presented the greatest suitability were in fact the municipalities with the highest incidence.

**MAIN CONCLUSIONS:**

The multicriteria analysis revealed that the elements that were used are suited for and consistent in the prediction of the areas that are vulnerable to SYF. The results obtained indicate the proximity of the areas that are most vulnerable to the disease to densely populated areas where an *Aedes aegypti* infestation was observed, which confers a high risk of re-urbanisation of YF.

The recent Yellow Fever (YF) outbreak in Brazil is the greatest in the history of the disease, which is a public health threat due to the considerable lethality of the disease, with an average mortality rate of 50%.^1^ ^,^ ^2^ In 2017, 718 cases were confirmed with 125 deaths attributed to the sylvatic transmission cycle.^3^ In this cycle, the YF virus (YFV) uses non-human primates as hosts and the main vectors are the mosquitoes of the *Haemagous* and *Sabethes* genera. This cycle is unlike the urban YF cycle, in which the human with YF is the host and one of the main vectors is *Aedes aegypti*.^1^

In Brazil, no cases of urban YF have been reported for 80 years, but with the increase in sylvatic Yellow Fever (SYF), the *Ae. aegypti* infestation in urban areas, and low vaccine coverage, urban YF has an increased introduction risk.^2^ By estimating the density of *Ae. aegypti* in some districts of the city of Rio de Janeiro, it was possible to verify that the presence of least one human infected with the YFV confers a risk of urban YF.^4^ The consequences, in addition to human suffering, are economic losses due to infeasibility of work of the patients, the costs of assistance, and reduction of tourism and exports.^1^

For disease control, a preventive vaccination is indispensable; however, what is observed in practice is the adoption of vaccination campaigns after the first case of YF in humans is reported. This is not a recommended strategy and might cause a reduction in vaccine supply as observed during the YF outbreak in Angola in 2015, in which the doses of the vaccine had to be fractionated to meet the needs of the entire population.^5^ Preventive vaccination is one of the measures established by the World Health Organization (WHO) to eliminate the occurrence of YF in humans by 2026.^6^

To this end, control measures are being progressively implemented^6^ and it is necessary to determine the areas at greater risk, which must be prioritised in the distribution of resources and implementation of vaccination campaigns. In addition, identifying the most susceptible regions is essential to establish prior vaccination of travellers that might act in the spread of the disease, including to other countries.^7^ To map the areas at risk, it is necessary to identify the factors related to the disease and to use the techniques and tools that assist the joint data analysis.

Geographical information systems (GIS) are highlighted for this function, since they allow for association of the cases of YF to geographical and socioeconomic characteristics through various integrative functions, which allows for identification of a pattern or tendency. Due to these characteristics, the GIS are commonly used in disease mapping and epidemiological studies.^7^ ^,^ ^8^

GIS application can provide indications of possible causes of SYF outbreak in Brazil since these are still unknown, with 224 cases reported in areas that were not considered susceptible to the disease.^3^ ^,^ ^5^ ^,^ ^9^ Understanding the causes allow to identify new areas amenable to the disease in which control and prevention measurements must be established. Thus, the objective of this study was to correlate the physical-environmental elements associated with the occurrence of SYF in humans by the means of a multicriteria analysis.

## MATERIALS AND METHODS

**Fig. 1:**
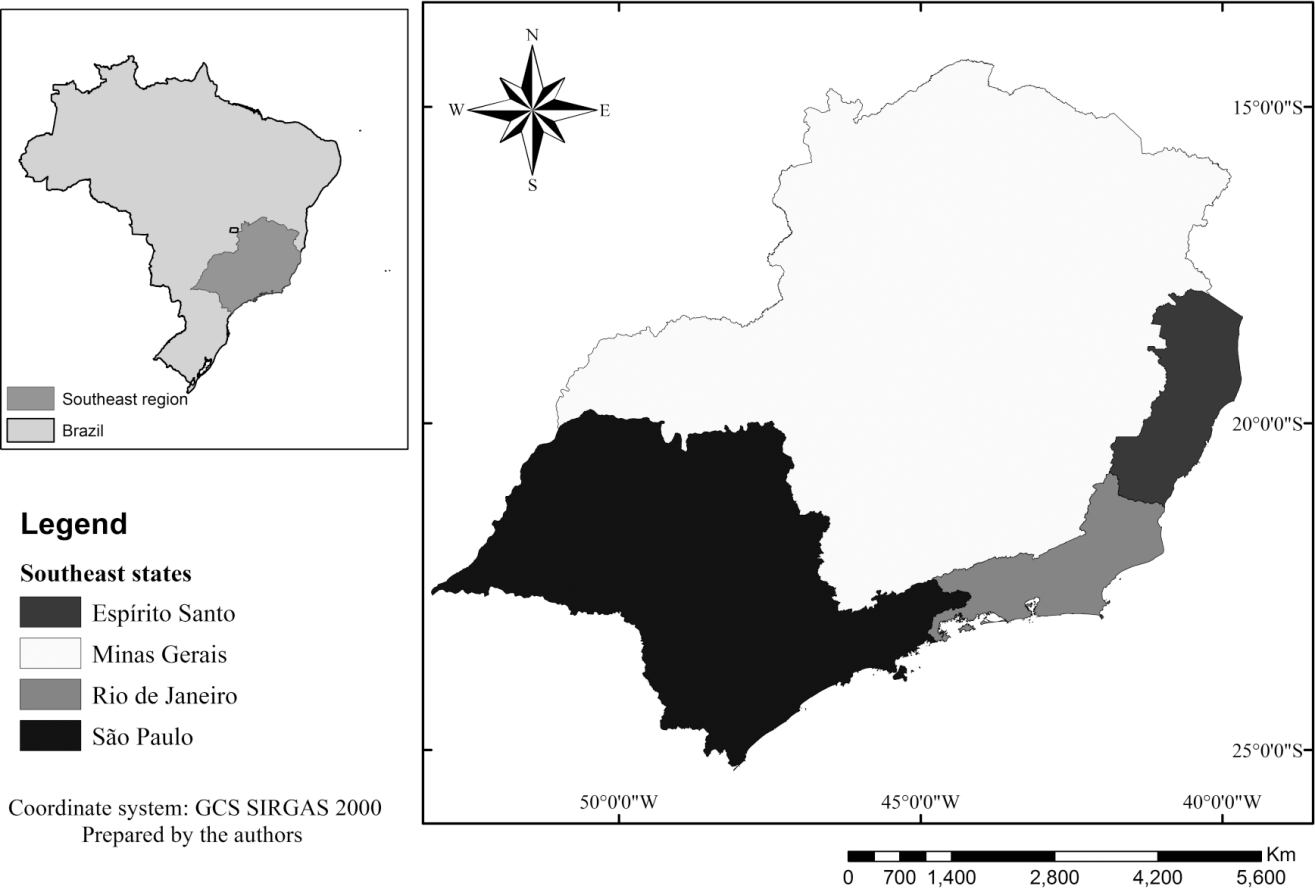
states in the southeast of Brazil.

*Area of study* - The Southeast region of Brazil is formed by the states of Minas Gerais, Rio de Janeiro, São Paulo, and Espírito Santo (Fig. 1) and it presents climates Aw, Af, BSh, and Cfa according to the Köppen climate classification^10^ with predominance of hot and rainy summers and dry winters. The region is of great economic importance to the country, housing approximately 80 million people (42% of the Brazilian population) with an average population density of 86.92 inhabitants by square kilometer.^11^

This region presents features typical of developing countries, such as a high population density and income concentration.

The occurrence of YF in humans in southeastern Brazil ensued via the sylvatic cycle and is higher than that in other regions of the country. In 2017, 125 deaths related to the disease were reported, while the combined number of YF-related deaths in the other regions was only three.^3^ It is important to note that in previous vulnerability maps, some southeastern states, such as Espírito Santo and Rio de Janeiro, were not considered to be susceptible to the disease^5^ ^,^ ^9^; however, some cases of the disease were reported in these states,^3^ which reiterates the need for investigation of the causes and elements related to the outbreak in this region.

*Multicriteria analysis and evaluation criteria for areas vulnerable to SYF* - The spatial analysis conducted to obtain the SYF vulnerability map was performed via multicriteria evaluation using Idrisi Selva^®^17.1 software. The multicriteria analysis environment of this software allows for the aggregation of the physical-environmental factors related to disease occurrence, namely non-human primates susceptible to the disease virus, vector presence, temperature, rainfall, altitude, and land cover and use.^5^ ^,^ ^7^ ^,^ ^9^ ^,^ ^12^

The geographical distribution of the non-human primates that are the most vulnerable to the YFV, *Alouatta* and *Callithrix*, was obtained from the Atlas of Brazilian Biodiversity available on the Chico Mendes Institute for Biodiversity Conservation (ICMBio) website, linked to the Ministry of Environment (http://www.icmbio.gov.br/portal/faunabrasileira/lista-de-especies?start=550). The Atlas presents the geographical distribution of the species of Brazilian fauna, with each species represented by a polygonal location in the national territory.

In the Southeast region, the following species that are notable reservoirs of the YFV were identified: *Alouatta guariba guariba*, *Alouatta guariba clamitans*, *Callithrix aurita*, and *Callithrix flaviceps*. In order to convert the distribution map of the *Alouatta* and *Callithrix* species to a format suitable for the multicriteria analysis, the image was initially georeferenced and transformed into a polygon consisting of the region where at least one of the non-human primate species was located, and the map was subsequently converted into a Boolean data type raster. This process of georeferencing and conversion to a raster file was performed using ArcMap^®^10.3 and Idrisi software (the files are available as Supplementary data I).

With regard to mosquitoes transmitting SYF, the following species were considered: *Haemagogus janthinomys* Dyar; *Haemogogus* (*Conopostegus leucocelanus* Dyar and Shannon), *Haemagogus spegazzinii* Brethes, *Haemagogus janthinomys*, *Sabethes chloropterus*, and *Sabethes cyaneus*.^13^ The prevalence of these species was obtained from the Global Biodiversity Information Facility (https://www.gbif.org/), which provides the geographical coordinates where the species in question were found. These coordinates were plotted and converted into a format suitable for performing the multicriteria analysis, and these procedures were performed using ArcMap and Idrisi software.

Unlike the raster used to represent the presence of nonhuman primates, consisting of a polygon, the raster representing mosquitoes transmitting SYF consists of points and may not represent the actual distribution of these species due to their ability to fly long distances. For this reason, and because the mosquito species *Haemagogus* and *Sabethes* are known to be present in forests in the Southeast region,^2^ these vectors were considered to be present in the forest areas. This consideration allows the inclusion of species with the potential to transmit the YFV, such as the *Aedes serratus* in the multicriteria analysis.^13^ ^,^ ^14^

Temperature and rainfall measurements at a spatial resolution of approximately 1 km were obtained on the electronic address of the Chelsa climate.^15^ The precipitation and temperature data provided by Chelsa refer to the mean values of these variables between 1979 and 2013.

The altitude was extracted from the digital elevation model with spatial resolution of approximately 1 km, obtained from the Global Multi-resolution Terrain Elevation Data 2010.^16^ Land cover and use data were obtained from the MapBiomas platform (http://mapbiomas.org/map#coverage), of the Greenhouse Effect Gas Emission System, which maps land cover and use in Brazil. The land use map used in this study refers to the year of 2017.

Southeast Brazil comprises three spindles of the Universal Transverse Mercator coordinate system; thus, in order to remedy possible distortions and measurement mistakes caused by the positioning of the region, a change in projection was performed using the ArcMap in order to maintain the distances. The projection that fit best was the Conic Equidistant Projection. Table I presents the main elements related to SYF along with their justification and influence limits.

**TABLE I:** Elements considered on the multicriteria analysis

Element	Justification	Reference
Altitude	The transmission of Yellow Fever (YF) can occur at altitudes of up to 2300 metres in the Americas.	Hamrick et. al.^7^; Kraemer et al.^17^
Rainfall	Exceeds 2000 mm. Climatic factors like moisture and temperature influence the abundance of vectors of the YF and the multiplication of the virus.	Hamlet et al^.(^ ^18^; Hamrick et. al.^7^; Kraemer et al.^17^
Temperature	0ºC to 30ºC	Hamlet et al.^18^; Hamrick et. al.^7^; Kraemer et al.^17^
Usage and occupation	Forests are the regions where the virus of sylvatic Yellow Fever (SYF).	Kraemer et al.^17^
Forest distance	The vector can be found in the areas nearby the forests and some species that transmits YF are apt to fly up to 11000 m.	Consoli and Lourenço-de-Oliveira^19^
Existence of non-human primates of the genera *Alouatta* and *Callithrix*	Sensitive to the virus and with high mortality rate.	ICMBio^20^; Hamrick et. al.^7^
Mosquitoes	The vectors that transmit the YF are of the family Culicidae, it is usually does not disperse more than 1000 m, however some species that transmits YF are apt to fly up to 11000 m.	Montagnera et al.^21^; Consoli and Lourenço-de-Oliveira^19^

After establishment of the elements involved in SYF occurrence, the factors used in the analysis were determined; that is, the criteria that attribute some degree of adequacy to the studied region according to the range of 0 (least adequacy) to 255 (most adequacy). Fuzzy functions were used to represent the factors and standardise them on the same scale of adequacy. Table II presents the functions and their respective control points according to the restrictions presented in Table I.

**TABLE II:** Factors associated with vulnerability criteria and their weights

Descrição	Função fuzzy	Control point				Weights
		a	b	c	d		
Forest distance	Decreasing sigmoidal	-	-	0	11000	0.2986
Existence of non-human primates *Alouatta* and *Callithrix*	Decreasing sigmoidal	-	-	0	11000	0.2986
Mosquitoes	Decreasing sigmoidal	-	-	1000	11000	0.1310
Temperature	Symmetric	0	10	30	50^*^	0.0563
Rainfall	Increasing sigmoidal	0	2000	-	-	0.0563
Altitude	Decreasing sigmoidal	-	-	2300	3000^*^	0.0283
Land cover and use	Agriculture 20 Savanna formation 150 Water courses 100 Planted forests 200 Forest formation 255 Grasslands 40 Urban area 1 Grove 150 Grasslands 40 Country vegetation 120	0 - 255 scale				0.1310

*: maximum value data.

Once the fuzzy functions were defined, each factor was weighted through the Analytical Hierarchical Process (Table III). This process consists of assigning a scale of importance between the factors analysed and comparing them in pairs in a matrix with a nine-point scale ranging from 1-9. A score of 1 indicates “much less important”, a score of 9 indicates “much more important”, and a middle score indicates “equally important”^8^ (Table III). To evaluate the consistency of the weight, it is necessary to observe the consistence relation (CR), which must be inferior to 0.10 to the weights to be considered consistent and usable.^22^

The importance assignment phase according to the comparator scale is considered one of the most important steps in the definition of risk areas, since the values awarded to a factor directly impact the obtained result. The weights attributed to the physical-environmental elements used in this study were defined according to the importance conferred by several health secretaries on the factors related to YF occurrence,^23^ and the factors of the existence of non-human primates *Alouatta* and *Callithrix*, presence of mosquitoes, and land cover and use with forest formations were awarded the greatest importance. Transitioning biomes, such as savanna formation and groves were awarded intermediate importance and the urban area category was awarded the lowest value in the analysis, since the present study aimed to analyse the sylvatic form of YF.

**TABLE III:** Association of factors in analytical hierarchical process (AHP)

Factors	Forest	Existence of non-human primates	Land cover and use	Mosquitoes	Temperature	Rainfall	Altitude
Forest	1						
Existence of non-human primates	1	1					
Land cover and use	1/3	1/3	1				
Mosquitoes	1/3	1/3	1	1			
Temperature	1/5	1/5	1/3	1/3	1		
Rainfall	1/5	1/5	1/3	1/3	1	1	
Altitude	1/7	1/7	1/5	1/5	1/3	1/3	1

1/5: strongly less important than; 1/3: moderately less important than; 1: equally important that.

The existence of non-human primates and forests were considered to be determining factors in the occurrence of YF because, as mentioned previously, the location of mosquitoes transmitting YF are geographical points and may not represent their real distribution. The mosquitoes in question inhabit the forests; we therefore believe that the forest element better represents the distribution of mosquitoes as transmitters of SYF. To avoid overlapping of importance, the elements of land cover and use and the presence of mosquitoes received lower weights. The temperature and precipitation factors received lower weights because they act as factors that favour the occurrence of the disease, along with altitude.

*Weighted linear combination* - Later, the weighted linear combination (WCL) method was applied to aggregate the criteria, due to its ability to attribute weights to each of the factors to allow for compensation. After the factors with low weight were compensated for by factors with high weight,^22^ we could identify the areas with maximum aptitude for the factors analysed.

*Validation* - To verify whether the vulnerability map was consistent with the reported cases of SYF in humans, the incidence of the disease in 100,000 was overlapped with the vulnerability map. The recorded cases of SYF in humans were obtained from the portal of the Ministry of Health (http://www2.datasus.gov.br/DATASUS/index.php?area=02) and population data were obtained from the portal of the Brazilian Institute of Geography and Statistics (https://www.ibge.gov.br/). The Regress statistical tool from Idrisi was also applied, which allows for the analysis of the relationship between two images by means of a regression analysis. Regression analysis is commonly used to describe the relationship between variables and can be evaluated by means of the correlation (r) and determination (R²) coefficients, with high values in both coefficients indicating a strong relationship. The map of the incidence of the disease was adopted as an independent variable and as a dependent variable of the SYF vulnerability map.

## RESULTS

**Fig. 2:**
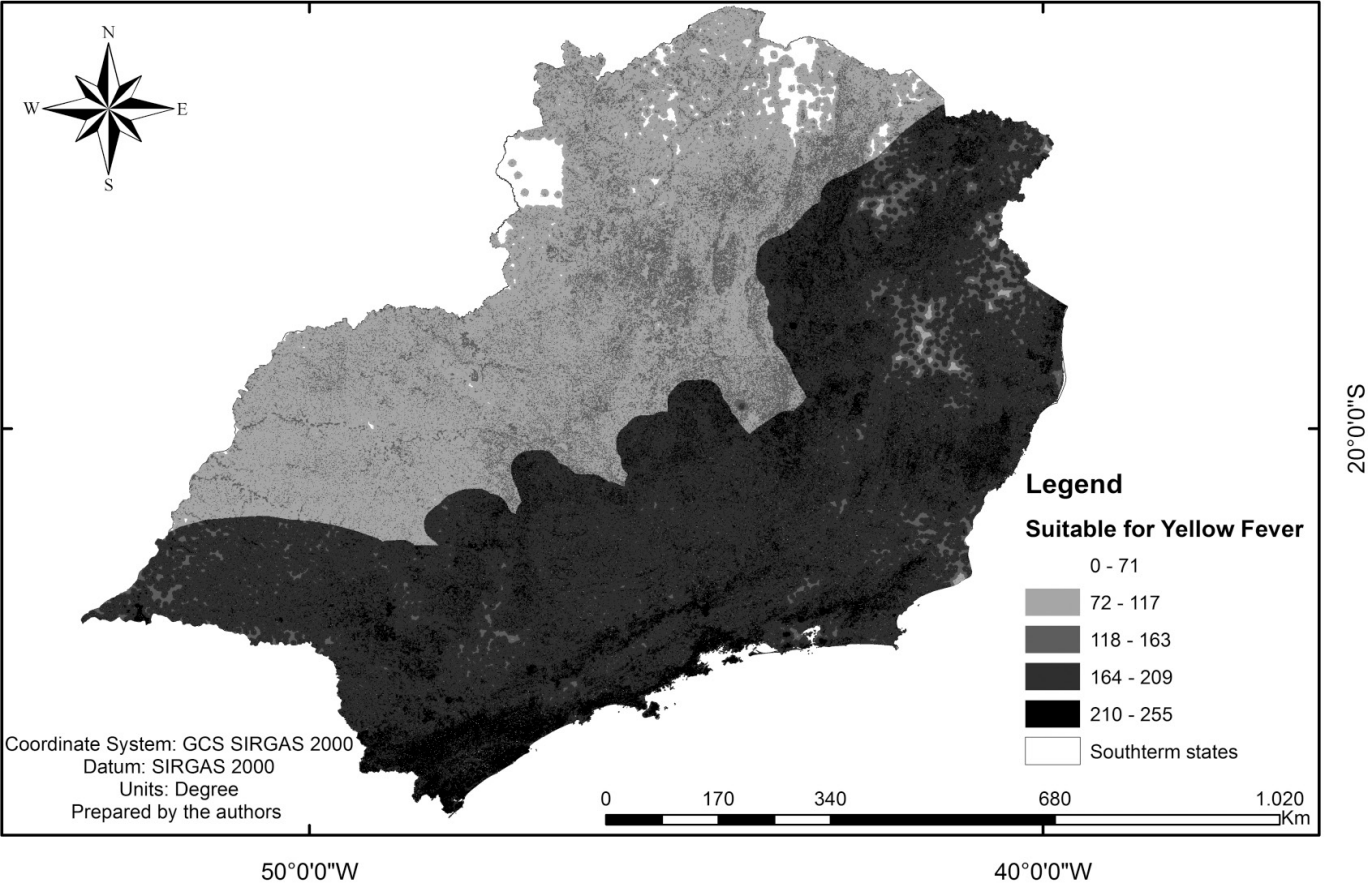
sylvatic Yellow Fever (SYF) suitability map in southeast Brazil.

Considering the importance attributed to each factor related to the occurrence of SYF in humans, the areas in the Southeast region that were most vulnerable to the disease were identified (Fig. 2). In the Northwest regions of São Paulo and Minas Gerais, the predominant suitability was <200, and in the states of Rio de Janeiro and Espírito Santo, it was > 200. In general, all states presented fragments of high suitability, with values reaching 255 on a 0 to 255 scale.

The multicriteria analysis identified three distinct areas regarding SYF occurrence suitability; one was a more susceptible region (suitability between 200 and 255), another was an intermediary susceptible region (suitability between 70 and 200), and another was a less susceptible region (suitability between 0 and 70).

The analysis indicated that parts of the regions northwest and north of Minas Gerais presented low vulnerability. However, some regions exhibited high adequacy, namely, the regions south of the state of São Paulo, northeast of Minas Gerais, and the states of Rio de Janeiro and Espirito Santo. It should be noted that regions most susceptible to the occurrence of SYF were located near the most densely populated areas, such as the metropolitan regions of São Paulo, Rio de Janeiro, and Belo Horizonte.

**Fig. 3:**
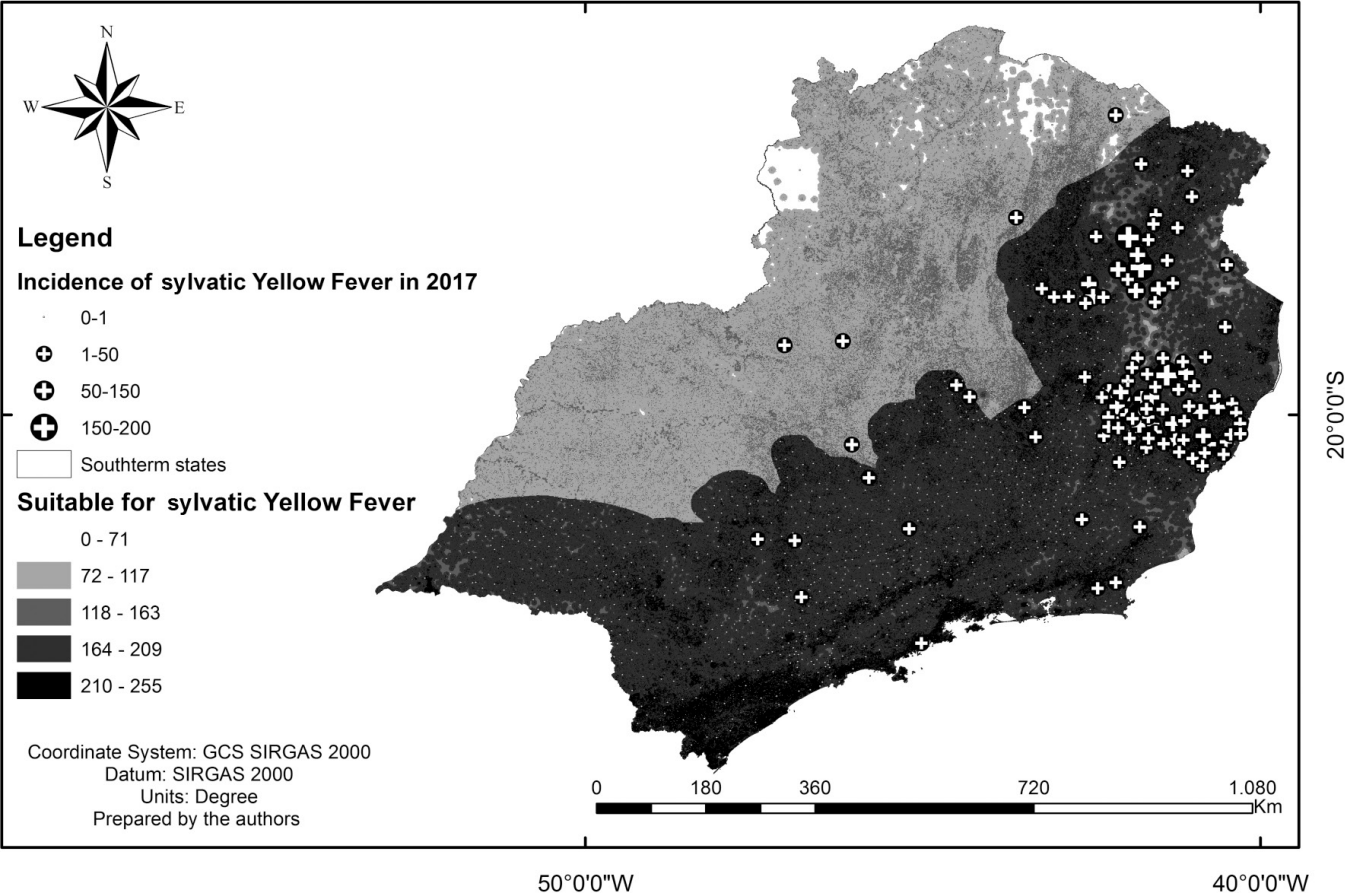
comparison of the suitability map and southeast Brazil municipalities with the incidence of sylvatic Yellow Fever (SYF) in humans in 2017.

To verify the representativeness of the vulnerability map obtained by the multicriteria analysis, the cities with highest incidence of YF were superimposed over the suitability map (Fig. 3).

The map of SYF vulnerability coincided with the areas in which cases of the disease have been recorded. The regions that presented the greatest suitability were the municipalities with the highest incidence of the disease in humans, with emphasis on the municipalities to the east and northeast in the state of Minas Gerais and to the south of Espírito Santo, where an incidence of > 23 cases of YF/100,000 inhabitants was noted. The state of Minas Gerais was the only region that presented cases in areas with a suitability of < 200. However, São Paulo was the only state that presented a region with high vulnerability without any recorded cases of YF in humans.

## DISCUSSION

The SYF vulnerability map for the Southeast region indicated two distinct regions with high and low adequacy. This distinction was due to the greater importance attributed to the factors that directly influence the existence of vectors, such as the forest category and their distance, as well as the existence of non-human primates susceptible to the virus and the presence of transmitting mosquitoes. These factors resulted in the states of Rio de Janeiro and Espirito Santo being associated with a high risk of the occurrence of SYF. It is important to note that Espírito Santo and Rio de Janeiro are fully within the Atlantic Forest domain, and Minas Gerais and São Paulo are also partially contained within this domain.

The Atlantic Forest is one of the largest tropical rainforests of the South American continent, home to a diversity of endemic species. It is also one of the most devastated forests, with loss of area and consequently of habitat, which favours the dispersion of mosquito transmitters. Another aspect that favours the dispersion of the YFV is the high flow of merchandise and human movement that occurs in the Southeast region due to the presence of one of the greatest urban centres in Brazil. This fact may explain the occurrence of the disease in regions that presented low vulnerability, as observed for the state of Minas Gerais.

In relation to the climatic factors, it was observed that temperature and precipitation were not factors that presented significant restriction, because the amplitude of both parameters that favoured SYF occurrence coincides with the averages observed for the studied region. That is, the mean temperature and mean precipitation in the Southeast region are high and adequate for the occurrence of YF throughout the region; thus, it was not possible to identify regions where these parameters act as a restriction. It is important to emphasize that the climatic elements are fundamental in the definition of the geographical patterns of SYF occurrence since they directly affect the vectors’ life cycles, as high precipitation and temperature increase the number of mosquitoes and larvae and consequently favour viral circulation. Altitude was also not a factor of high restriction, because the transmitting mosquitoes can be found at diverse altitudes.

When observing the overlap of the incidences of SYF on the vulnerability map (Fig. 3), the southern region of the state of São Paulo presented high adequacy, but no cases of the disease were reported. The existence of various state and federal conservation units conferred high suitability. It is believed that no cases of the disease were reported because the areas are of restricted access and receive vaccination recommendation.^24^ Recent notifications have confirmed that the highest number of deaths of non-human primates due to the YFV occurred in the state of São Paulo,^25^ which is consistent with the results obtained in this study. The areas with high suitability near the metropolitan region of São Paulo may confer high potential for re-urbanisation of YF. For the other states, the results indicate the proximity of the most vulnerable areas of SYF to densely populated areas where an *Ae. aegypti* infestation is observed, which also confers a high risk of YF re-urbanisation.

With the exception of the southern region of the state of São Paulo, the most susceptible areas identified in the multicriteria analysis coincided with the areas in which SYF in humans was recorded, indicating that the factors, weights and amounts, control points, and fuzzy functions adopted in this study were consistent and adequate. In addition, the weights used were consistent and usable with a CR of 0.02, which is within the recommended value (< 0.1) and the Regress tool provided a correlation coefficient (r) of 0.86 and a determination coefficient (R^2^) of 0.74. These values indicate that there is a strong correlation between the reported cases of SYF in humans and the areas of greater suitability present in the vulnerability map.

To identify the areas subject to the occurrence of SYF in humans, several factors were correlated (land cover and use, existence of non-human primates, mosquitoes, forest distance, temperature, rainfall, and altitude) by means of sophisticated tools and procedures that were later validated. However, a limitation of this study and object for future research is the inclusion of factors related to population vaccination and records of occurrence of YFV in non-human primates.

The addition of vaccine coverage information to the multicriteria analysis may contribute to the greater refinement of areas vulnerable to YF, since knowledge of areas already immunised can annul some of the physical-environmental factors. Epizootics monitoring (YF in non-human primates) indicates areas with viral circulation and the risk of occurrence of the disease in humans.

We overlapped the occurrence of the YFV in non-human primates (provided by Faria et al.^26^) and in humans^3^ to the vulnerability map of the state of Minas Gerais [the table with cases of the disease in humans, and both images are available as Supplementary data II (Figs 1-2, Table)]. The results indicated that 80.5% and 98.7% of the number of cases recorded in non-human primates and in humans, respectively, occurred in the most vulnerable area (adequacy between 200 and 241, with 241 being the maximum suitability for the state). These findings again indicated that the most vulnerable areas identified in this study have viral circulation.

Despite these results, epizootic cases of YF have been reported in the northwestern and southwestern regions of the state of Minas Gerais.^26^ These regions present intermediate vulnerability, which indicates that the map obtained in this study should be used as an aid for the adoption of preventive measures, but these actions should not be restricted to the regions of greater adequacy since YF can present high dynamics, with a reported viral dispersion of 4.25 km/day.^26^

*In conclusion* - Temporal analysis of YF in Southeast Brazil allowed us to infer that several factors contributed to the occurrence of the outbreak in 2017, including low vaccine coverage and environmental imbalance in the vectors’ habitat. The vulnerability map generated in the multicriteria analysis made it possible to identify that a large portion of Rio de Janeiro and Espírito Santo and considerable parts of Minas Gerais and São Paulo are risk areas that should receive the public power’s attention in order to develop more effective vaccination strategies.

The factors and weights adopted in this study proved to be relevant to the reported cases of the disease; this result reiterates the importance of non-human primates and vector mosquitoes when defining areas at risk of YF occurrence. The regression analysis for the vulnerability map validation indicated that the areas that were most vulnerable to the occurrence of the YF are the places in which the most cases are reported.

The prediction of vulnerable areas to the disease via spatial analysis proved to be an important tool for epidemiologic studies, which can be used as an instrument for the formation of vaccination policies as a method to prevent outbreaks and to minimise the adverse events of the disease.
