# Supplementary material for: Determination of the spatial susceptibility to Yellow Fever using a multicriteria analysis
Source: Mem Inst Oswaldo Cruz. 2019 May 6;114:e180509. doi: 10.1590/0074-02760180509 (PMC6506150; doi:10.1590/0074-02760180509)
Supplement: Supplementary file 2 [file 1678-8060-mioc-114-e180509-s2.pdf]

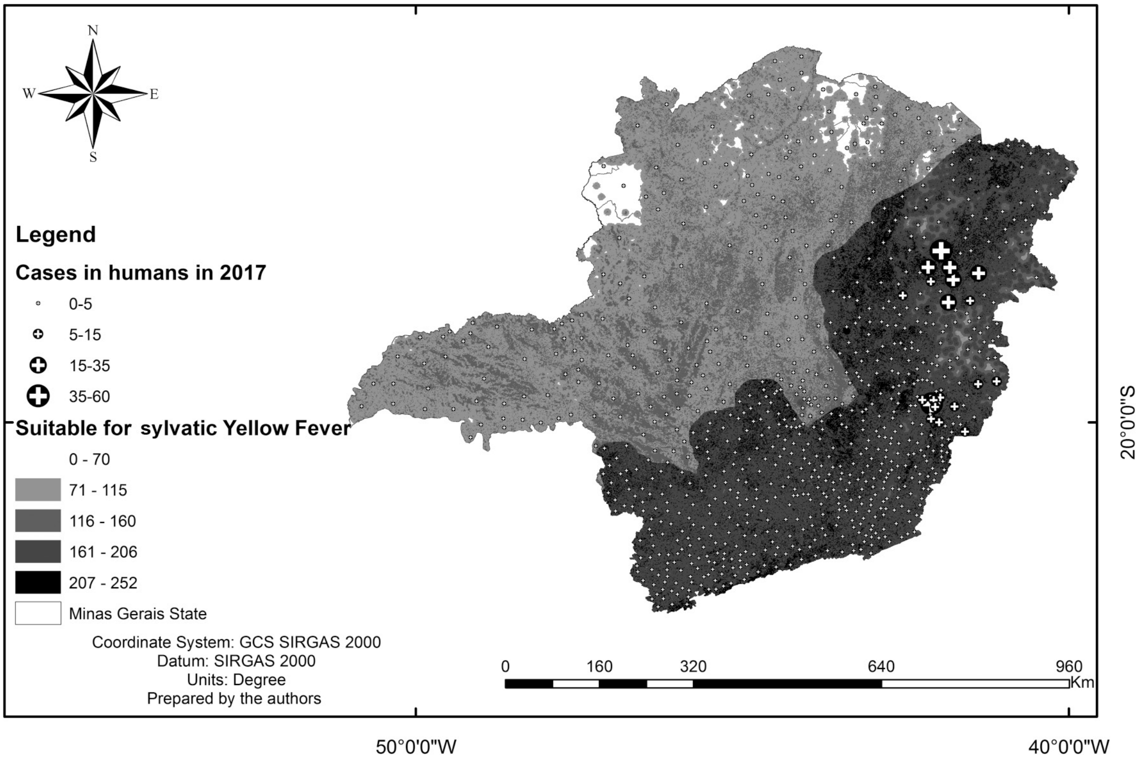

Fig. 1: comparison of the suitability map and cases of sylvatic yellow fever (SYF) in humans in 2017.

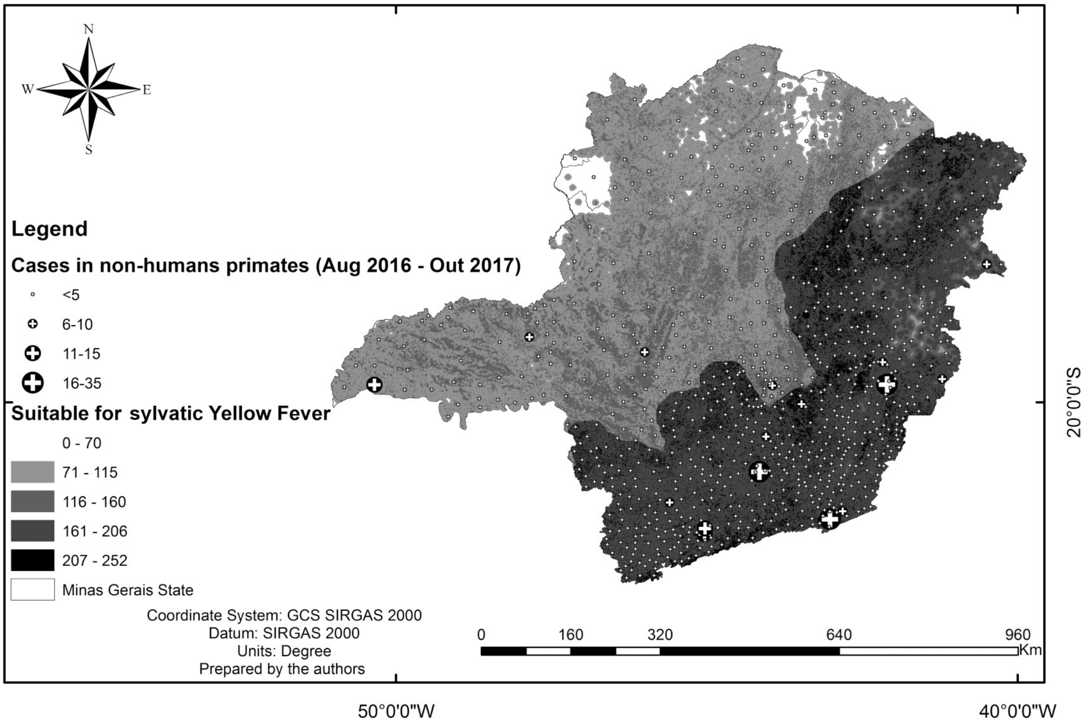

Fig. 2: comparison of the suitability map and cases of sylvatic yellow fever (SYF) in non-human primates in 2017.

CC BY

Coordinates Human Yellow Fever data

COD_IBGE	City	State of Brasil	Cases of Yellow Fever in 2017	Incidence of Yellow Fever in 2017 (Number of cases per 100000 inhabitant)	Geographic coordinates (SIRGAS 2000)	
					Latitude	Longitude
3157609	Santa Fé de Minas	MG	0.0	0.00	-16.70210075	-45.55799866
3550803	São Sebastião da Grama	SP	0.0	0.00	-2.175390053	-46.75360107
3157658	Santa Helena de Minas	MG	0.0	0.00	-16.9041996	-4.065919876
3550902	São Simão	SP	0.0	0.00	-21.46179962	-47.5685997
3157708	Santa Juliana	MG	0.0	0.00	-19.38159943	-47.50930023
3551009	São Vicente	SP	0.0	0.00	-23.95890045	-4.648920059
3157807	Santa Luzia	MG	0.0	0.00	-19.74559975	-43.83750153
3551108	Sarapuí	SP	0.0	0.00	-23.66419983	-47.78609848
3157906	Santa Margarida	MG	0.0	0.00	-20.42840004	-4.226699829
3551207	Sarutaiá	SP	0.0	0.00	-23.25749969	-49.48220062
3158003	Santa Maria de Itabira	MG	0.0	0.00	-19.42869949	-4.306399918
3551306	Sebastianópolis do Sul	SP	0.0	0.00	-2.062969971	-49.91590118
3158102	Santa Maria do Salto	MG	0.0	0.00	-16.30879974	-40.11790085
3551405	Serra Azul	SP	0.0	0.00	-21.31620026	-47.54999924
3158201	Santa Maria do Suaçuí	MG	4.0	2.78	-18.25849915	-42.32550049
3551504	Serrana	SP	0.0	0.00	-21.2173996	-47.6128006
3158300	Santana da Vargem	MG	0.0	0.00	-21.27149963	-45.5060997
3551603	Serra Negra	SP	0.0	0.00	-22.58919907	-46.68650055
3158409	Santana de Cataguases	MG	0.0	0.00	-21.28339958	-42.55709839
3551702	Sertãozinho	SP	0.0	0.00	-21.12339973	-48.00920105
3158508	Santana de Pirapama	MG	0.0	0.00	-18.91930008	-43.90520096
3551801	Sete Barras	SP	0.0	0.00	-24.27429962	-47.93560028
3158607	Santana do Deserto	MG	0.0	0.00	-21.94650078	-43.18399811
3551900	Severínia	SP	0.0	0.00	-2.079380035	-4.879380035
3158706	Santana do Garambéu	MG	0.0	0.00	-21.63820076	-44.06689835
3158805	Santana do Jacaré	MG	0.0	0.00	-20.88400078	-45.06370163
3552007	Silveiras	SP	0.0	0.00	-22.73489952	-44.84120178
3552106	Socorro	SP	0.0	0.00	-22.61149979	-46.52470016
3158904	Santana do Manhuaçu	MG	2.0	2.33	-20.05690002	-41.89319992
3552205	Sorocaba	SP	0.0	0.00	-2.346479988	-4.744710159
3158953	Santana do Paraíso	MG	0.0	0.00	-19.38500023	-42.52159882
3552304	Sud Mennucci	SP	0.0	0.00	-20.66729927	-50.90119934
3159001	Santana do Riacho	MG	0.0	0.00	-1.918969917	-43.64599991
3552403	Sumaré	SP	0.0	0.00	-22.84160042	-47.2635994
3159100	Santana dos Montes	MG	0.0	0.00	-20.79789925	-43.67070007
3552502	Suzano	SP	0.0	0.00	-23.60910034	-46.3105011
3159209	Santa Rita de Caldas	MG	0.0	0.00	-22.02470016	-46.27230072
3552551	Suzanápolis	SP	0.0	0.00	-20.47389984	-51.07580185
3159308	Santa Rita de Jacutinga	MG	0.0	0.00	-22.09980011	-44.09640121
3552601	Tabapuã	SP	0.0	0.00	-20.93199921	-4.902949905
3159357	Santa Rita de Minas	MG	4.0	6.11	-19.87529945	-42.12469864
3552700	Tabatinga	SP	0.0	0.00	-21.71360016	-48.63100052
3159407	Santa Rita de Ibitipoca	MG	0.0	0.00	-21.57839966	-4.393109894
3552809	Taboão da Serra	SP	0.0	0.00	-23.62039948	-46.7867012
3159506	Santa Rita do Itueto	MG	9.0	1.58	-19.41379929	-41.38970184
3552908	Taciba	SP	0.0	0.00	-22.50499916	-51.34249878
3159605	Santa Rita do Sapucaí	MG	0.0	0.00	-22.23789978	-45.68360138
3553005	Taguaí	SP	0.0	0.00	-23.47400093	-49.39770126
3159704	Santa Rosa da Serra	MG	0.0	0.00	-19.56220055	-46.00439835
3159803	Santa Vitória	MG	0.0	0.00	-18.9904995	-50.27640152
3553104	Taiaçu	SP	0.0	0.00	-21.13199997	-48.5306015
3553203	Taiúva	SP	0.0	0.00	-21.13269997	-48.42869949
3159902	Santo Antônio do Amparo	MG	0.0	0.00	-20.91790009	-44.94440079
3553302	Tambaú	SP	0.0	0.00	-21.6019001	-47.23910141
3160009	Santo Antônio do Aventureiro	MG	0.0	0.00	-2.175219917	-4.28125E-05
3553401	Tanabi	SP	0.0	0.00	-20.53140068	-49.64699936
3160108	Santo Antônio do Grama	MG	0.0	0.00	-20.32250023	-4.260480118
3553500	Tapiraí	SP	0.0	0.00	-24.00880051	-47.61949921
3160207	Santo Antônio do Itambé	MG	0.0	0.00	-18.47929955	-43.26900101
3553609	Tapiratiba	SP	0.0	0.00	-21.45100021	-46.74229813
3160306	Santo Antônio do Jacinto	MG	0.0	0.00	-1.65053997	-40.2784996
3553658	Taquaral	SP	0.0	0.00	-21.0692997	-48.39970016
3160405	Santo Antônio do Monte	MG	0.0	0.00	-20.08429909	-45.29259872
3553708	Taquaritinga	SP	0.0	0.00	-21.42580032	-48.53839874
3160454	Santo Antônio do Retiro	MG	0.0	0.00	-15.28950024	-42.66139984
3553807	Taquarituba	SP	0.0	0.00	-23.53019905	-49.23500061
3160504	Santo Antônio do Rio Abaixo	MG	0.0	0.00	-19.24189949	-43.25030136
3553856	Taquarivaí	SP	0.0	0.00	-23.94339943	-48.68859863
3160603	Santo Hipólito	MG	0.0	0.00	-18.39100075	-0.044173199
3553906	Tarabai	SP	0.0	0.00	-2.235950089	-51.62689972
3160702	Santos Dumont	MG	0.0	0.00	-21.45870018	-43.52679825
3553955	Tarumã	SP	0.0	0.00	-2.276329994	-50.60189819
3160801	São Bento Abade	MG	0.0	0.00	-21.57069969	-45.07310104
3554003	Tatuí	SP	0.0	0.00	-23.36219978	-47.86989975
3160900	São Brás do Suaçuí	MG	0.0	0.00	-2.062820053	-43.97320175
3554102	Taubaté	SP	0.0	0.00	-23.08729935	-45.50270081
3160959	São Domingos das Dores	MG	0.0	0.00	-19.52540016	-42.03070068
3554201	Tejupá	SP	0.0	0.00	-23.3423996	-49.30690002
3161007	São Domingos do Prata	MG	0.0	0.00	-19.90399933	-4.290449905
3554300	Teodoro Sampaio	SP	0.0	0.00	-22.41699982	-52.37440109
3161056	São Félix de Minas	MG	0.0	0.00	-18.56999969	-41.44850159
3554409	Terra Roxa	SP	0.0	0.00	-20.77890015	-48.3465004
3161106	São Francisco	MG	0.0	0.00	-15.89729977	-44.86289978

CC BY

3554508	Tietê	SP	0.0	0.00	-23.0503006	-47.70920181
3161205	São Francisco de Paula	MG	0.0	0.00	-20.71769905	-44.99649811
3161304	São Francisco de Sales	MG	0.0	0.00	-19.79750061	-49.85960007
3554607	Timburi	SP	0.0	0.00	-23.19560051	-49.61109924
3554656	Torre de Pedra	SP	0.0	0.00	-23.25040054	-48.21300125
3161403	São Francisco do Glória	MG	0.0	0.00	-20.79269981	-42.28340149
3554706	Torrinha	SP	0.0	0.00	-22.44350052	-48.16419983
3161502	São Geraldo	MG	0.0	0.00	-20.91139984	-42.83000183
3554755	Trabiju	SP	0.0	0.00	-22.03249931	-48.34600067
3161601	São Geraldo da Piedade	MG	0.0	0.00	-18.89699936	-42.31119919
3554805	Tremembé	SP	0.0	0.00	-22.9409008	-45.60409927
3161650	São Geraldo do Baixio	MG	0.0	0.00	-18.91720009	-4.136740112
3554904	Três Fronteiras	SP	0.0	0.00	-20.27269936	-50.86999893
3161700	São Gonçalo do Abaeté	MG	0.0	0.00	-18.17819977	-4.554700089
3554953	Tuiuti	SP	0.0	0.00	-22.83110046	-46.68799973
3161809	São Gonçalo do Pará	MG	0.0	0.00	-1.998450089	-0.448297005
3555000	Tupã	SP	0.0	0.00	-21.94790077	-50.53540039
3161908	São Gonçalo do Rio Abaixo	MG	0.0	0.00	-19.81839943	-43.32239914
3555109	Tupi Paulista	SP	0.0	0.00	-21.39069939	-51.58769989
3162005	São Gonçalo do Sapucaí	MG	0.0	0.00	-21.91189957	-45.59500122
3555208	Turiúba	SP	0.0	0.00	-20.94099998	-50.11130142
3162104	São Gotardo	MG	0.0	0.00	-19.3579998	-45.99919891
3555307	Turmalina	SP	0.0	0.00	-20.08399963	-50.45740128
3162203	São João Batista do Glória	MG	0.0	0.00	-20.57780075	-46.43989944
3555356	Ubarana	SP	0.0	0.00	-21.21980095	-4.974850082
3162252	São João da Lagoa	MG	0.0	0.00	-16.86190033	-44.33599854
3162302	São João da Mata	MG	0.0	0.00	-21.94309998	-45.92440033
3555505	Ubirajara	SP	0.0	0.00	-22.54389954	-49.66630173
3162401	São João da Ponte	MG	0.0	0.00	-15.90950012	-43.89609909
3555604	Uchoa	SP	0.0	0.00	-20.93759918	-4.915800095
3162450	São João das Missões	MG	0.0	0.00	-14.89260006	-44.22869873
3555703	União Paulista	SP	0.0	0.00	-20.89109993	-49.88740158
3162500	São João del Rei	MG	0.0	0.00	-21.26379967	-44.27700043
3555802	Urânia	SP	0.0	0.00	-20.20910072	-50.65280151
3162559	São João do Manhuaçu	MG	0.0	0.00	-2.037030029	-42.15439987
3555901	Uru	SP	0.0	0.00	-2.176650047	-49.29719925
3162575	São João do Manteninha	MG	0.0	0.00	-18.75209999	-41.16469955
3556008	Urupês	SP	0.0	0.00	-21.20490074	-49.26530075
3162609	São João do Oriente	MG	0.0	0.00	-19.35409927	-42.17129898
3556107	Valentim Gentil	SP	0.0	0.00	-20.42020035	-50.10229874
3162658	São João do Pacuí	MG	0.0	0.00	-16.55669975	-44.52479935
3556206	Valinhos	SP	0.0	0.00	-22.97559929	-46.98160172
3162708	São João do Paraíso	MG	0.0	0.00	-15.34370041	-41.97750092
3556305	Valparaíso	SP	0.0	0.00	-21.20350075	-50.92480087
3162807	São João Evangelista	MG	0.0	0.00	-18.51460075	-4.277659988
3556354	Vargem	SP	0.0	0.00	-22.88999939	-46.41479874
3162906	São João Nepomuceno	MG	0.0	0.00	-21.58399963	-43.00429916
3556404	Vargem Grande do Sul	SP	2.0	0.51	-21.86059952	-46.89899826
3162922	São Joaquim de Bicas	MG	0.0	0.00	-20.07119942	-44.24959946
3556453	Vargem Grande Paulista	SP	0.0	0.00	-23.62829971	-47.01660156
3162948	São José da Barra	MG	0.0	0.00	-20.74990082	-46.24489975
3556503	Várzea Paulista	SP	0.0	0.00	-23.21980095	-4.682479858
3162955	São José da Lapa	MG	0.0	0.00	-19.69720078	-43.99079895
3556602	Vera Cruz	SP	0.0	0.00	-22.23430061	-49.82659912
3163003	São José da Safira	MG	0.0	0.00	-18.31780052	-42.12419891
3556701	Vinhedo	SP	0.0	0.00	-23.04669952	-46.97919846
3163102	São José da Varginha	MG	0.0	0.00	-19.6970005	-44.56240082
3556800	Viradouro	SP	0.0	0.00	-20.88430023	-48.31029892
3163201	São José do Alegre	MG	0.0	0.00	-22.33099937	-45.51900101
3556909	Vista Alegre do Alto	SP	0.0	0.00	-2.117670059	-48.64960098
3163300	São José do Divino	MG	0.0	0.00	-18.39929962	-4.137670136
3556958	Vitória Brasil	SP	0.0	0.00	-20.19869995	-5.048600006
3163409	São José do Goiabal	MG	0.0	0.00	-19.93309975	-42.69580078
3557006	Votorantim	SP	0.0	0.00	-23.58029938	-47.4070015
3163508	São José do Jacuri	MG	0.0	0.00	-18.24360085	-42.66930008
3557105	Votuporanga	SP	0.0	0.00	-20.45809937	-49.99010086
3163607	São José do Mantimento	MG	4.0	1.54	-20.02099991	-41.77119827
3557154	Zacarias	SP	0.0	0.00	-21.12520027	-50.04999924
3163706	São Lourenço	MG	0.0	0.00	-22.11759949	-45.03499985
3557204	Chavantes	SP	0.0	0.00	-23.04660034	-49.72700119
3163805	São Miguel do Anta	MG	0.0	0.00	-2.072739983	-42.71179962
3557303	Estiva Gerbi	SP	0.0	0.00	-22.23649979	-46.94290161
3163904	São Pedro da União	MG	0.0	0.00	-2.112890053	-46.64289856
3164001	São Pedro dos Ferros	MG	0.0	0.00	-20.05520058	-42.57049942
3164100	São Pedro do Suaçuí	MG	1.0	1.80	-18.34869957	-42.59389877
3164209	São Romão	MG	0.0	0.00	-16.39019966	-4.540769959
3164308	São Roque de Minas	MG	0.0	0.00	-20.18799973	-4.651399994
3164407	São Sebastião da Bela Vista	MG	0.0	0.00	-22.16230011	-45.77669907
3164431	São Sebastião da Vargem Alegre	MG	0.0	0.00	-21.02490044	-42.60039902
3164472	São Sebastião do Anta	MG	0.0	0.00	-19.50810051	-41.95759964
3164506	São Sebastião do Maranhão	MG	12.0	1.13	-18.05990028	-42.54169846
3164605	São Sebastião do Oeste	MG	0.0	0.00	-20.25349998	-45.04470062
3164704	São Sebastião do Paraíso	MG	0.0	0.00	-20.9246006	-47.00680161
3164803	São Sebastião do Rio Preto	MG	0.0	0.00	-19.30120087	-43.22100067
3164902	São Sebastião do Rio Verde	MG	0.0	0.00	-22.22039986	-45.02529907
3165008	São Tiago	MG	0.0	0.00	-20.93959999	-44.5617981
3165107	São Tomás de Aquino	MG	0.0	0.00	-20.78709984	-47.12789917

3165206	São Thomé das Letras	MG	0.0	0.00	-21.7343998	-44.96490097
3165305	São Vicente de Minas	MG	0.0	0.00	-21.66819954	-44.4756012
3165404	Sapucaí-Mirim	MG	0.0	0.00	-22.78700066	-45.84899902
3165503	Sardoá	MG	0.0	0.00	-18.77560043	-42.40790176
3165537	Sarzedo	MG	0.0	0.00	-20.05999947	-44.1216011
3165552	Setubinha	MG	32.0	2.94	-17.62290001	-42.15710068
3165560	Sem-Peixe	MG	0.0	0.00	-20.08069992	-42.82600021
3165578	Senador Amaral	MG	0.0	0.00	-22.55850029	-46.21770096
3165602	Senador Cortes	MG	0.0	0.00	-21.77149963	-42.90990067
3165701	Senador Firmino	MG	0.0	0.00	-20.90539932	-43.10570145
3165800	Senador José Bento	MG	0.0	0.00	-2.215769959	-46.14229965
3165909	Senador Modestino Gonçalves	MG	0.0	0.00	-17.8560009	-43.24169922
3166006	Senhora de Oliveira	MG	0.0	0.00	-20.80200005	-43.35039902
3166105	Senhora do Porto	MG	0.0	0.00	-18.91390038	-43.08160019
3166204	Senhora dos Remédios	MG	0.0	0.00	-21.03219986	-4.360139847
3166303	Sericita	MG	0.0	0.00	-20.49290085	-42.45949936
3166402	Seritinga	MG	0.0	0.00	-21.91740036	-44.46030045
3166501	Serra Azul de Minas	MG	0.0	0.00	-18.39209938	-43.20840073
3166600	Serra da Saudade	MG	0.0	0.00	-19.3864994	-45.77870178
3166709	Serra dos Aimorés	MG	0.0	0.00	-17.78479958	-40.26490021
3166808	Serra do Salitre	MG	0.0	0.00	-19.15970039	-46.65520096
3166907	Serrania	MG	0.0	0.00	-21.55520058	-46.09360123
3166956	Serranópolis de Minas	MG	0.0	0.00	-15.8572998	-42.86259842
3167004	Serranos	MG	0.0	0.00	-21.8321991	-44.53789902
3167103	Serro	MG	0.0	0.00	-18.53709984	-43.4029007
3167202	Sete Lagoas	MG	0.0	0.00	-19.43770027	-44.25450134
3167301	Silveirânia	MG	0.0	0.00	-21.1446991	-43.20429993
3167400	Silvianópolis	MG	0.0	0.00	-22.03770065	-45.80670166
3167509	Simão Pereira	MG	0.0	0.00	-21.96509933	-43.29399872
3167707	Sobrália	MG	0.0	0.00	-19.21820068	-42.14680099
3167806	Soledade de Minas	MG	0.0	0.00	-22.02779961	-45.0284996
3167905	Tabuleiro	MG	0.0	0.00	-21.36429977	-43.25690079
3168002	Taiobeiras	MG	0.0	0.00	-15.82289982	-42.05369949
3168051	Taparuba	MG	2.0	6.38	-19.73880005	-41.61190033
3168101	Tapira	MG	0.0	0.00	-19.91650009	-46.86940002
3168200	Tapiraí	MG	0.0	0.00	-19.87739944	-4.616239929
3168309	Taquaraçu de Minas	MG	0.0	0.00	-19.63470078	-43.68669891
3168408	Tarumirim	MG	1.0	7.00	-19.30190086	-41.90259933
3168507	Teixeiras	MG	0.0	0.00	-20.63279915	-42.86339951
3168606	Teófilo Otoni	MG	27.0	2.00	-17.71570015	-41.38380051
3168705	Timóteo	MG	0.0	0.00	-19.55830002	-42.60350037
3168804	Tiradentes	MG	0.0	0.00	-21.11790085	-44.15570068
3168903	Tiros	MG	0.0	0.00	-18.86149979	-45.82509995
3169000	Tocantins	MG	0.0	0.00	-21.17880058	-43.02730179
3169059	Tocos do Moji	MG	0.0	0.00	-22.35919952	-46.14789963
3169109	Toledo	MG	0.0	0.00	-2.270619965	-46.38349915
3169208	Tombos	MG	0.0	0.00	-2.088290024	-42.06560135
3169307	Três Corações	MG	1.0	1.37	-21.68709946	-45.20589828
3169356	Três Marias	MG	0.0	0.00	-18.29899979	-45.06999969
3169406	Três Pontas	MG	0.0	0.00	-21.39439964	-45.49919891
3169505	Tumiritinga	MG	0.0	0.00	-19.0156002	-41.70729828
3169604	Tupaciguara	MG	0.0	0.00	-18.53580093	-48.75619888
3169703	Turmalina	MG	0.0	0.00	-17.2451992	-42.84090042
3169802	Turvolândia	MG	0.0	0.00	-21.88570023	-45.80080032
3169901	Ubá	MG	0.0	0.00	-21.10659981	-42.96620178
3170057	Ubaporanga	MG	7.0	5.81	-19.65999985	-42.07089996
3170206	Uberlândia	MG	0.0	0.00	-1.902879906	-48.33209991
3170305	Umburatiba	MG	0.0	0.00	-17.27389908	-40.66680145
3170404	Unaí	MG	0.0	0.00	-16.37639999	-46.81999969
3170438	União de Minas	MG	0.0	0.00	-19.40970039	-50.34370041
3170479	Uruana de Minas	MG	0.0	0.00	-16.09609985	-46.31949997
3170503	Urucânia	MG	0.0	0.00	-20.32620049	-4.273109818
3170529	Urucuia	MG	0.0	0.00	-16.0284996	-45.57450104
3170578	Vargem Alegre	MG	0.0	0.00	-19.60440063	-42.32239914
3170602	Vargem Bonita	MG	0.0	0.00	-2.04321003	-46.33679962
3170651	Vargem Grande do Rio Pardo	MG	0.0	0.00	-15.3416996	-42.29970169
3170701	Varginha	MG	0.0	0.00	-2.155859947	-45.41009903
3170750	Varjão de MInas	MG	0.0	0.00	-18.46750069	-45.93619919
3170800	Várzea da Palma	MG	0.0	0.00	-1.749679947	-44.72710037
3170909	Varzelândia	MG	0.0	0.00	-15.64410019	-43.93610001
3171006	Vazante	MG	0.0	0.00	-17.86980057	-46.86840057
3171030	Verdelândia	MG	0.0	0.00	-15.55200005	-43.63399887
3171071	Veredinha	MG	0.0	0.00	-17.48810005	-42.7256012
3171105	Veríssimo	MG	0.0	0.00	-19.60300064	-48.34090042
3171154	Vermelho Novo	MG	2.0	0.43	-20.03470039	-42.25510025
3171204	Vespasiano	MG	0.0	0.00	-19.73220062	-43.9457016
3171303	Viçosa	MG	0.0	0.00	-20.74090004	-42.88669968
3171402	Vieiras	MG	0.0	0.00	-20.91270065	-4.228269959
3171501	Mathias Lobato	MG	0.0	0.00	-18.6114006	-41.92829895
3171600	Virgem da Lapa	MG	0.0	0.00	-16.71610069	-42.34049988
3171709	Virgínia	MG	0.0	0.00	-2.234300041	-4.510950089
3171808	Virginópolis	MG	0.0	0.00	-18.7928009	-42.67110062
3171907	Virgolândia	MG	0.0	0.00	-18.45190048	-42.31829834
3172004	Visconde do Rio Branco	MG	0.0	0.00	-21.0128994	-42.8409996
3172103	Volta Grande	MG	0.0	0.00	-21.76000023	-42.55899811
3172202	Wenceslau Braz	MG	0.0	0.00	-22.55559921	-45.40269852
3167608	Simonésia	MG	6.0	3.28	-19.99950027	-41.98789978

3129707	Ibiraci	MG	0.0	0.00	-20.39649963	-47.1108017
3139003	Machado	MG	0.0	0.00	-21.66699982	-45.92089844
3170107	Uberaba	MG	0.0	0.00	-19.58139992	-47.97719955
3170008	Ubaí	MG	0.0	0.00	-16.36499977	-44.84939957
3117876	Confins	MG	0.0	0.00	-19.64319992	-43.97570038
3154903	Rio Casca	MG	0.0	0.00	-20.14170074	-42.66149902
3200102	Afonso Cláudio	ES	11.0	3.54	-20.08930016	-41.12879944
3200136	Águia Branca	ES	0.0	0.00	-18.97159958	-40.74980164
3200169	Água Doce do Norte	ES	0.0	0.00	-18.5189991	-40.99629974
3200201	Alegre	ES	0.0	0.00	-20.72030067	-41.51290131
3200300	Alfredo Chaves	ES	5.0	3.58	-20.56480026	-40.82740021
3200359	Alto Rio Novo	ES	0.0	0.00	-19.02199936	-40.98749924
3200409	Anchieta	ES	0.0	0.00	-20.7201004	-40.68709946
3200508	Apiacá	ES	0.0	0.00	-21.07290077	-41.55469894
3200607	Aracruz	ES	0.0	0.00	-19.7682991	-4.017639923
3200706	Atilio Vivacqua	ES	0.0	0.00	-20.9640007	-41.18830109
3200805	Baixo Guandu	ES	9.0	3.09	-19.57069969	-40.98339844
3200904	Barra de São Francisco	ES	0.0	0.00	-18.66659927	-40.83129883
3201001	Boa Esperança	ES	0.0	0.00	-18.48550034	-40.32820129
3201100	Bom Jesus do Norte	ES	0.0	0.00	-21.08499908	-41.63439941
3201159	Brejetuba	ES	8.0	6.71	-20.12989998	-41.29899979
3201209	Cachoeiro de Itapemirim	ES	1.0	0.00	-2.076869965	-41.18989944
3201308	Cariacica	ES	9.0	2.58	-20.29120064	-40.44340134
3201407	Castelo	ES	4.0	1.15	-2.055170059	-41.20439911
3201506	Colatina	ES	1.0	0.00	-19.48579979	-40.65610123
3201605	Conceição da Barra	ES	0.0	0.00	-18.44569969	-39.83359909
3201704	Conceição do Castelo	ES	8.0	6.85	-20.37059975	-41.26750183
3201803	Divino de São Lourenço	ES	0.0	0.00	-20.58779907	-41.72499847
3201902	Domingos Martins	ES	26.0	8.16	-20.30830002	-40.84930038
3202009	Dores do Rio Preto	ES	0.0	0.00	-20.64159966	-41.81259918
3202108	Ecoporanga	ES	0.0	0.00	-18.2656002	-4.080780029
3202207	Fundão	ES	1.0	5.87	-19.9673996	-40.36259842
3202306	Guaçuí	ES	0.0	0.00	-20.76440048	-41.7057991
3202454	Ibatiba	ES	8.0	3.58	-20.25110054	-41.55059814
3202504	Ibiraçu	ES	1.0	8.95	-19.83539963	-40.41889954
3202553	Ibitirama	ES	3.0	3.35	-20.48620033	-41.6935997
3202603	Iconha	ES	1.0	7.99	-20.75569916	-40.85810089
3202652	Irupi	ES	2.0	1.71	-20.33180046	-41.6371994
3202702	Itaguaçu	ES	0.0	0.00	-19.72800064	-40.86520004
3202801	Itapemirim	ES	0.0	0.00	-20.9666996	-40.94670105
3202900	Itarana	ES	7.0	6.43	-19.94939995	-40.88890076
3203007	Iúna	ES	3.0	1.10	-20.34889984	-41.6570015
3203056	Jaguaré	ES	0.0	0.00	-18.9503994	-40.00429916
3203106	Jerônimo Monteiro	ES	0.0	0.00	-20.8132	-41.39360046
3203130	João Neiva	ES	0.0	0.00	-19.71249962	-40.43439865
3203163	Laranja da Terra	ES	1.0	9.24	-19.87319946	-41.05830002
3203205	Linhares	ES	0.0	0.00	-19.38269997	-40.02909851
3203304	Mantenópolis	ES	0.0	0.00	-18.87669945	-41.07239914
3203320	Marataízes	ES	0.0	0.00	-21.10050011	-40.89319992
3203346	Marechal Floriano	ES	18.0	1.26	-20.43169975	-40.7745018
3203353	Marilândia	ES	0.0	0.00	-19.43589973	-40.51919937
3203403	Mimoso do Sul	ES	0.0	0.00	-21.08889961	-41.37779999
3203502	Montanha	ES	0.0	0.00	-18.13929939	-40.2757988
3203601	Mucurici	ES	0.0	0.00	-18.01530075	-40.51300049
3203700	Muniz Freire	ES	9.0	4.89	-20.42760086	-41.43560028
3203809	Muqui	ES	0.0	0.00	-20.93849945	-41.34540176
3203908	Nova Venécia	ES	1.0	2.17	-18.69949913	-40.52370071
3204005	Pancas	ES	2.0	9.28	-19.14489937	-40.81840134
3204054	Pedro Canário	ES	0.0	0.00	-1.81821003	-40.0381012
3204104	Pinheiros	ES	0.0	0.00	-18.36969948	-40.20069885
3204203	Piúma	ES	0.0	0.00	-20.83839989	-40.77349854
3204252	Ponto Belo	ES	0.0	0.00	-18.25040054	-40.51490021
3204302	Presidente Kennedy	ES	0.0	0.00	-21.14340019	-41.07310104
3204351	Rio Bananal	ES	0.0	0.00	-1.923130035	-4.030929947
3204401	Rio Novo do Sul	ES	0.0	0.00	-20.81909943	-40.9192009
3204500	Santa Leopoldina	ES	26.0	2.12	-20.12369919	-40.54029846
3204559	Santa Maria de Jetibá	ES	0.0	0.00	-20.08569908	-4.080459976
3204609	Santa Teresa	ES	13.0	5.96	-1.987820053	-40.63600159
3204658	São Domingos do Norte	ES	0.0	0.00	-19.12299919	-40.56679916
3204708	São Gabriel da Palha	ES	0.0	0.00	-18.95319939	-40.50899887
3204807	São José do Calçado	ES	0.0	0.00	-20.98340034	-41.65629959
3204906	São Mateus	ES	0.0	0.00	-18.74909973	-40.02149963
3204955	São Roque do Canaã	ES	1.0	8.87	-19.72039986	-40.68009949
3205002	Serra	ES	11.0	2.69	-2.012890053	-40.30160141
3205010	Sooretama	ES	0.0	0.00	-19.07229996	-40.14810181
3205036	Vargem Alta	ES	3.0	1.57	-20.64699936	-41.00460052
3205069	Venda Nova do Imigrante	ES	4.0	1.96	-20.37129974	-41.14009857
3205101	Viana	ES	2.0	3.08	-20.40169907	-40.51369858
3205150	Vila Pavão	ES	0.0	0.00	-18.61450005	-40.62760162
3205176	Vila Valério	ES	0.0	0.00	-18.96680069	-4.033140183
3300159	Aperibé	RJ	0.0	0.00	-21.65390015	-4.212990189
3300209	Araruama	RJ	1.0	0.00	-22.75569916	-42.29389954
3300225	Areal	RJ	0.0	0.00	-22.23590088	-43.12269974
3300233	Armação dos Búzios	RJ	0.0	0.00	-22.7765007	-41.95100021
3300258	Arraial do Cabo	RJ	0.0	0.00	-22.92259979	-42.16989899
3300308	Barra do Piraí	RJ	0.0	0.00	-22.42690086	-43.91379929
3300407	Barra Mansa	RJ	0.0	0.00	-22.50670052	-44.18889999

3300456	Belford Roxo	RJ	0.0	0.00	-22.72929955	-43.37799835
3300506	Bom Jardim	RJ	0.0	0.00	-22.20089912	-42.37310028
3300605	Bom Jesus do Itabapoana	RJ	0.0	0.00	-21.12400055	-41.68389893
3300704	Cabo Frio	RJ	1.0	0.00	-22.71039963	-42.05770111
3300803	Cachoeiras de Macacu	RJ	0.0	0.00	-22.51670074	-42.72919846
3300902	Cambuci	RJ	0.0	0.00	-21.49720001	-41.91490173
3300936	Carapebus	RJ	0.0	0.00	-22.20120049	-41.63499832
3300951	Comendador Levy Gasparian	RJ	0.0	0.00	-22.04400063	-43.25669861
3301009	Campos dos Goytacazes	RJ	0.0	0.00	-21.74690056	-41.40499878
3301108	Cantagalo	RJ	0.0	0.00	-21.86919975	-42.33470154
3301157	Cardoso Moreira	RJ	0.0	0.00	-21.52529907	-41.50239944
3301207	Carmo	RJ	0.0	0.00	-21.90239906	-42.56919861
3301306	Casimiro de Abreu	RJ	6.0	1.70	-22.48259926	-42.14500046
3301405	Conceição de Macabu	RJ	0.0	0.00	-22.13999939	-41.82889938
3301504	Cordeiro	RJ	0.0	0.00	-22.05890083	-42.34270096
3301603	Duas Barras	RJ	0.0	0.00	-22.05699921	-42.49890137
3301702	Duque de Caxias	RJ	0.0	0.00	-22.63190079	-43.30049896
3301801	Engenheiro Paulo de Frontin	RJ	0.0	0.00	-22.51819992	-43.63909912
3301850	Guapimirim	RJ	0.0	0.00	-22.58370018	-42.96519852
3301876	Iguaba Grande	RJ	0.0	0.00	-2.282900047	-42.21760178
3301900	Itaboraí	RJ	0.0	0.00	-22.75040054	-42.85620117
3302056	Italva	RJ	0.0	0.00	-2.142569923	-41.65470123
3302106	Itaocara	RJ	0.0	0.00	-2.172809982	-42.08330154
3302205	Itaperuna	RJ	0.0	0.00	-21.22509956	-4.1895401
3302254	Itatiaia	RJ	0.0	0.00	-22.43939972	-4.458440018
3302270	Japeri	RJ	0.0	0.00	-22.6609993	-43.60680008
3302304	Laje do Muriaé	RJ	0.0	0.00	-21.25110054	-42.13660049
3302452	Macuco	RJ	0.0	0.00	-22.02540016	-42.2737999
3302502	Magé	RJ	0.0	0.00	-22.61300087	-43.11399841
3302700	Maricá	RJ	0.0	0.00	-22.91600037	-42.81809998
3302809	Mendes	RJ	0.0	0.00	-22.53170013	-4.374850082
3302908	Miguel Pereira	RJ	0.0	0.00	-22.5067997	-43.46910095
3303005	Miracema	RJ	0.0	0.00	-21.39609909	-42.15269852
3303104	Natividade	RJ	0.0	0.00	-21.04190063	-41.93170166
3303203	Nilópolis	RJ	0.0	0.00	-22.82130051	-43.42979813
3303302	Niterói	RJ	0.0	0.00	-22.91659927	-43.05590057
3303401	Nova Friburgo	RJ	0.0	0.00	-22.32019997	-42.5019989
3303500	Nova Iguaçu	RJ	1.0	0.00	-22.68950081	-43.50220108
3303609	Paracambi	RJ	0.0	0.00	-22.62220001	-43.72610092
3303708	Paraíba do Sul	RJ	0.0	0.00	-2.218429947	-43.30630112
3303856	Paty do Alferes	RJ	0.0	0.00	-22.36660004	-43.40579987
3303906	Petrópolis	RJ	0.0	0.00	-22.40250015	-43.16159821
3303955	Pinheiral	RJ	0.0	0.00	-22.54260063	-43.99990082
3304003	Piraí	RJ	0.0	0.00	-22.6345005	-43.9048996
3304102	Porciúncula	RJ	0.0	0.00	-20.90690041	-41.96760178
3304110	Porto Real	RJ	0.0	0.00	-22.44169998	-44.3266983
3304128	Quatis	RJ	0.0	0.00	-22.35549927	-44.23749924
3304144	Queimados	RJ	0.0	0.00	-22.72550011	-43.58560181
3304151	Quissamã	RJ	0.0	0.00	-22.10499954	-41.4416008
3304201	Resende	RJ	0.0	0.00	-22.44169998	-44.4878006
3304300	Rio Bonito	RJ	0.0	0.00	-22.73590088	-42.58929825
3304409	Rio Claro	RJ	0.0	0.00	-2.278269959	-44.07939911
3304508	Rio das Flores	RJ	0.0	0.00	-22.16040039	-43.53979874
3304524	Rio das Ostras	RJ	0.0	0.00	-22.45490074	-41.94739914
3100104	Abadia dos Dourados	MG	0.0	0.00	-18.35400009	-47.45819855
3304607	Santa Maria Madalena	RJ	0.0	0.00	-2.197019959	-41.91350174
3100203	Abaeté	MG	0.0	0.00	-19.10420036	-45.36019897
3304706	Santo Antônio de Pádua	RJ	0.0	0.00	-21.55739975	-42.19309998
3100302	Abre Campo	MG	0.0	0.00	-20.2737999	-42.4435997
3304755	São Francisco de Itabapoana	RJ	0.0	0.00	-21.41760063	-41.13550186
3100401	Acaiaca	MG	0.0	0.00	-20.40110016	-43.10200119
3304805	São Fidélis	RJ	1.0	2.66	-21.66049957	-41.78820038
3100500	Açucena	MG	0.0	0.00	-19.05340004	-42.4510994
3100609	Água Boa	MG	0.0	0.00	-18.02799988	-42.27600098
3305000	São João da Barra	RJ	0.0	0.00	-21.78350067	-41.08069992
3100708	Água Comprida	MG	0.0	0.00	-2.00053997	-48.08459854
3305109	São João de Meriti	RJ	0.0	0.00	-22.78569984	-43.36619949
3100807	Aguanil	MG	0.0	0.00	-20.97540092	-45.4233017
3305133	São José de Ubá	RJ	0.0	0.00	-21.37100029	-41.94950104
3305158	São José do Vale do Rio Preto	RJ	0.0	0.00	-2.217740059	-42.93939972
3100906	Águas Formosas	MG	0.0	0.00	-17.04240036	-40.97460175
3305208	São Pedro da Aldeia	RJ	0.0	0.00	-22.78639984	-42.1242981
3101003	Águas Vermelhas	MG	0.0	0.00	-15.68459988	-41.52199936
3305307	São Sebastião do Alto	RJ	0.0	0.00	-21.88409996	-4.210309982
3101102	Aimorés	MG	1.0	4.01	-19.62599945	-41.21440125
3101201	Aiuruoca	MG	0.0	0.00	-2.195549965	-44.64849854
3305406	Sapucaia	RJ	0.0	0.00	-22.02529907	-4.282360077
3101300	Alagoa	MG	0.0	0.00	-22.18779945	-44.66189957
3305505	Saquarema	RJ	0.0	0.00	-22.87949944	-42.51950073
3101409	Albertina	MG	0.0	0.00	-22.19890022	-46.61370087
3305554	Seropédica	RJ	0.0	0.00	-22.75979996	-43.70330048
3101508	Além Paraíba	MG	0.0	0.00	-21.82760048	-42.74750137
3305604	Silva Jardim	RJ	2.0	9.37	-22.56830025	-42.4137001
3101607	Alfenas	MG	0.0	0.00	-21.39010048	-45.96709824
3305703	Sumidouro	RJ	0.0	0.00	-22.11330032	-4.266730118
3101631	Alfredo Vasconcelos	MG	0.0	0.00	-21.14080048	-43.71179962
3305752	Tanguá	RJ	0.0	0.00	-22.78129959	-4.272809982

3305802	Teresópolis	RJ	0.0	0.00	-22.31439972	-42.87409973
3101706	Almenara	MG	0.0	0.00	-16.09160042	-40.72109985
3305901	Trajano de Morais	RJ	0.0	0.00	-22.13069916	-42.15610123
3101805	Alpercata	MG	0.0	0.00	-18.98480034	-42.01959991
3101904	Alpinópolis	MG	0.0	0.00	-20.86109924	-4.637649918
3306008	Três Rios	RJ	0.0	0.00	-22.12470055	-43.11510086
3102001	Alterosa	MG	0.0	0.00	-21.2269001	-46.15370178
3306107	Valença	RJ	0.0	0.00	-22.23509979	-43.85839844
3102050	Alto Caparaó	MG	0.0	0.00	-20.45929909	-41.86579895
3306156	Varre-Sai	RJ	0.0	0.00	-20.89319992	-41.8266983
3306206	Vassouras	RJ	0.0	0.00	-22.36779976	-43.58190155
3102100	Alto Rio Doce	MG	0.0	0.00	-2.10366993	-43.40459824
3306305	Volta Redonda	RJ	0.0	0.00	-2.249139977	-44.08879852
3102209	Alvarenga	MG	2.0	0.05	-19.3973999	-41.67720032
3102308	Alvinópolis	MG	0.0	0.00	-20.11249924	-43.15359879
3102407	Alvorada de Minas	MG	0.0	0.00	-18.77969933	-43.36899948
3102506	Amparo do Serra	MG	0.0	0.00	-20.53009987	-42.80369949
3102605	Andradas	MG	0.0	0.00	-22.07659912	-46.55540085
3102704	Cachoeira de Pajeú	MG	0.0	0.00	-15.97439957	-41.50270081
3102803	Andrelândia	MG	0.0	0.00	-21.7397995	-44.27099991
3102852	Angelândia	MG	0.0	0.00	-17.70490074	-42.28310013
3102902	Antônio Carlos	MG	0.0	0.00	-21.4109993	-43.76660156
3103009	Antônio Dias	MG	0.0	0.00	-19.55870056	-42.88330078
3103108	Antônio Prado de Minas	MG	0.0	0.00	-21.02359962	-42.15269852
3103207	Araçaí	MG	0.0	0.00	-19.23500061	-44.22000122
3103306	Aracitaba	MG	0.0	0.00	-21.35129929	-4.340449905
3103405	Araçuaí	MG	0.0	0.00	-16.93099976	-41.99330139
3103504	Araguari	MG	0.0	0.00	-1.857729912	-48.22320175
3103603	Arantina	MG	0.0	0.00	-21.8973999	-44.22819901
3103702	Araponga	MG	0.0	0.00	-20.66320038	-42.49959946
3103751	Araporã	MG	0.0	0.00	-1.847739983	-49.12829971
3103801	Arapuá	MG	0.0	0.00	-19.04269981	-46.11560059
3103900	Araújos	MG	0.0	0.00	-19.88710022	-45.18190002
3104007	Araxá	MG	0.0	0.00	-19.63699913	-46.97299957
3104106	Arceburgo	MG	0.0	0.00	-21.36009979	-46.94210052
3104205	Arcos	MG	0.0	0.00	-20.26099968	-45.54790115
3104304	Areado	MG	0.0	0.00	-21.35720062	-46.1446991
3104403	Argirita	MG	0.0	0.00	-21.63619995	-42.83349991
3104452	Aricanduva	MG	0.0	0.00	-17.88159943	-4.260630035
3104502	Arinos	MG	0.0	0.00	-15.75010014	-45.98759842
3104601	Astolfo Dutra	MG	0.0	0.00	-21.31649971	-42.88029861
3104700	Ataléia	MG	0.0	0.00	-18.1644001	-41.15230179
3104809	Augusto de Lima	MG	0.0	0.00	-18.10980034	-44.19850159
3104908	Baependi	MG	0.0	0.00	-22.01790047	-4.482329941
3105004	Baldim	MG	0.0	0.00	-19.26429939	-43.86429977
3105103	Bambuí	MG	0.0	0.00	-2.011170006	-45.99969864
3105202	Bandeira	MG	0.0	0.00	-15.87849998	-40.58160019
3105301	Bandeira do Sul	MG	0.0	0.00	-21.72500038	-46.38619995
3105400	Barão de Cocais	MG	1.0	3.52	-19.89259911	-43.49639893
3105509	Barão de Monte Alto	MG	0.0	0.00	-21.26589966	-42.27930069
3105608	Barbacena	MG	0.0	0.00	-21.25270081	-43.79270172
3105707	Barra Longa	MG	0.0	0.00	-20.27680016	-43.06760025
3105905	Barroso	MG	0.0	0.00	-21.18449974	-43.95890045
3106002	Bela Vista de Minas	MG	0.0	0.00	-19.79899979	-43.1067009
3106101	Belmiro Braga	MG	0.0	0.00	-21.9864006	-43.4695015
3106200	Belo Horizonte	MG	5.0	0.00	-19.90329933	-43.96070099
3106309	Belo Oriente	MG	0.0	0.00	-19.25989914	-42.45100021
3106408	Belo Vale	MG	0.0	0.00	-20.42550087	-44.06110001
3106507	Berilo	MG	0.0	0.00	-16.8628006	-42.49520111
3106606	Bertópolis	MG	0.0	0.00	-16.97669983	-40.57509995
3106655	Berizal	MG	0.0	0.00	-1.568480015	-41.76200104
3106705	Betim	MG	2.0	0.00	-19.94799995	-44.19990158
3106804	Bias Fortes	MG	0.0	0.00	-21.62579918	-43.7743988
3106903	Bicas	MG	0.0	0.00	-21.73250008	-43.10599899
3107000	Biquinhas	MG	0.0	0.00	-18.75779915	-45.54000092
3107109	Boa Esperança	MG	0.0	0.00	-21.0496006	-45.62340164
3107208	Bocaina de Minas	MG	0.0	0.00	-22.2166996	-44.48400116
3107307	Bocaiúva	MG	0.0	0.00	-17.28860092	-43.6882019
3107406	Bom Despacho	MG	0.0	0.00	-1.968600082	-45.2879982
3107505	Bom Jardim de Minas	MG	0.0	0.00	-21.9477005	-44.12549973
3107604	Bom Jesus da Penha	MG	0.0	0.00	-21.01539993	-46.52999878
3107703	Bom Jesus do Amparo	MG	0.0	0.00	-19.71870041	-43.47029877
3107802	Bom Jesus do Galho	MG	4.0	2.60	-19.74270058	-42.35210037
3107901	Bom Repouso	MG	0.0	0.00	-22.44650078	-46.18489838
3108008	Bom Sucesso	MG	0.0	0.00	-21.02739906	-44.78540039
3108107	Bonfim	MG	0.0	0.00	-20.32209969	-44.21590042
3108206	Bonfinópolis de Minas	MG	0.0	0.00	-16.48830032	-46.20009995
3108255	Bonito de Minas	MG	0.0	0.00	-14.99569988	-44.89889908
3108305	Borda da Mata	MG	0.0	0.00	-22.25760078	-46.15280151
3108404	Botelhos	MG	0.0	0.00	-21.63759995	-0.464202995
3108503	Botumirim	MG	0.0	0.00	-16.93869972	-4.300640106
3108552	Brasilândia de Minas	MG	0.0	0.00	-16.95770073	-45.90319824
3108602	Brasília de Minas	MG	0.0	0.00	-16.24340057	-44.43980026
3108701	Brás Pires	MG	0.0	0.00	-20.87639999	-43.22859955
3108800	Braúnas	MG	0.0	0.00	-19.02630043	-42.71760178
3108909	Brasópolis	MG	0.0	0.00	-22.49419975	-45.62870026
3109006	Brumadinho	MG	0.0	0.00	-20.17849922	-44.12390137

3109105	Bueno Brandão	MG	0.0	0.00	-22.48699951	-46.35599899
3109204	Buenópolis	MG	0.0	0.00	-17.88299942	-44.04130173
3109253	Bugre	MG	0.0	0.00	-19.3586998	-42.31129837
3109303	Buritis	MG	0.0	0.00	-15.4538002	-46.60490036
3500105	Adamantina	SP	0.0	0.00	-21.57719994	-51.05709839
3109402	Buritizeiro	MG	0.0	0.00	-17.28720093	-45.19290161
3500204	Adolfo	SP	0.0	0.00	-21.28849983	-49.6534996
3109451	Cabeceira Grande	MG	0.0	0.00	-16.07679939	-47.12440109
3500303	Aguaí	SP	0.0	0.00	-22.05019951	-47.04040146
3109501	Cabo Verde	MG	0.0	0.00	-21.48259926	-46.39250183
3500402	Águas da Prata	SP	0.0	0.00	-21.9137001	-46.69229889
3109600	Cachoeira da Prata	MG	0.0	0.00	-19.51779938	-44.47309875
3500501	Águas de Lindóia	SP	0.0	0.00	-22.47480011	-46.60390091
3109709	Cachoeira de Minas	MG	0.0	0.00	-22.36330032	-45.79320145
3109808	Cachoeira Dourada	MG	0.0	0.00	-18.60790062	-49.48040009
3500550	Águas de Santa Bárbara	SP	0.0	0.00	-22.85890007	-49.26129913
3500600	Águas de São Pedro	SP	0.0	0.00	-2.260160065	-4.787670136
3109907	Caetanópolis	MG	0.0	0.00	-19.34329987	-44.4048996
3500709	Agudos	SP	0.0	0.00	-22.57180023	-49.10919952
3110004	Caeté	MG	0.0	0.00	-19.86870003	-43.63809967
3500758	Alambari	SP	0.0	0.00	-23.54899979	-47.86959839
3110103	Caiana	MG	0.0	0.00	-20.72669983	-41.90930176
3500808	Alfredo Marcondes	SP	0.0	0.00	-21.93519974	-51.39569855
3110202	Cajuri	MG	0.0	0.00	-20.78580093	-42.76509857
3500907	Altair	SP	0.0	0.00	-20.53339958	-49.0943985
3110301	Caldas	MG	0.0	0.00	-21.89159966	-46.36470032
3501004	Altinópolis	SP	0.0	0.00	-21.01810074	-47.38669968
3110400	Camacho	MG	0.0	0.00	-20.64179993	-45.1330986
3501103	Alto Alegre	SP	0.0	0.00	-21.61289978	-5.019240189
3110509	Camanducaia	MG	0.0	0.00	-22.78770065	-46.07580185
3501152	Alumínio	SP	0.0	0.00	-23.53319931	-0.472815018
3110608	Cambuí	MG	0.0	0.00	-22.58410072	-46.07960129
3501202	Álvares Florence	SP	0.0	0.00	-20.2901001	-49.9211998
3110707	Cambuquira	MG	0.0	0.00	-2.185950089	-45.26279831
3501301	Álvares Machado	SP	0.0	0.00	-22.13159943	-51.51520157
3110806	Campanário	MG	0.0	0.00	-18.2840004	-41.73500061
3501400	Álvaro de Carvalho	SP	0.0	0.00	-22.08650017	-49.73429871
3110905	Campanha	MG	0.0	0.00	-21.84469986	-4.540919876
3501509	Alvinlândia	SP	0.0	0.00	-22.45240021	-49.75899887
3111002	Campestre	MG	0.0	0.00	-21.71120071	-46.22240067
3111101	Campina Verde	MG	0.0	0.00	-19.47920036	-49.8105011
3501608	Americana	SP	0.0	0.00	-22.72360039	-47.28939819
3501707	Américo Brasiliense	SP	0.0	0.00	-21.72220039	-48.03290176
3111150	Campo Azul	MG	0.0	0.00	-16.50930023	-44.76409912
3501806	Américo de Campos	SP	0.0	0.00	-2.028989983	-49.76879883
3111200	Campo Belo	MG	0.0	0.00	-20.9211998	-45.25749969
3501905	Amparo	SP	2.0	3.04	-22.69890022	-46.79869843
3111309	Campo do Meio	MG	0.0	0.00	-21.1154995	-45.85089874
3111408	Campo Florido	MG	0.0	0.00	-19.71430016	-48.64749908
3502002	Analândia	SP	0.0	0.00	-22.12120056	-47.67829895
3502101	Andradina	SP	0.0	0.00	-20.83699989	-51.32989883
3111507	Campos Altos	MG	0.0	0.00	-19.60750008	-46.19609833
3502200	Angatuba	SP	0.0	0.00	-23.43700027	-48.45980072
3111606	Campos Gerais	MG	0.0	0.00	-21.25989914	-45.75429916
3502309	Anhembi	SP	0.0	0.00	-22.80039978	-4.817520142
3111705	Canaã	MG	0.0	0.00	-20.66769981	-42.62279892
3502408	Anhumas	SP	0.0	0.00	-22.36219978	-51.42829895
3111804	Canápolis	MG	0.0	0.00	-18.75849915	-49.27280045
3502507	Aparecida	SP	0.0	0.00	-22.9144001	-45.23759842
3111903	Cana Verde	MG	0.0	0.00	-2.103370094	-45.18619919
3502606	Aparecida d'Oeste	SP	0.0	0.00	-20.47920036	-50.92210007
3112000	Candeias	MG	0.0	0.00	-20.74959946	-45.28039932
3502705	Apiaí	SP	0.0	0.00	-24.4218998	-48.81940079
3112059	Cantagalo	MG	0.0	0.00	-18.51659966	-42.64929962
3502754	Araçariguama	SP	0.0	0.00	-23.43490028	-47.07279968
3112109	Caparaó	MG	0.0	0.00	-20.52840042	-41.9496994
3502804	Araçatuba	SP	0.0	0.00	-2.111420059	-50.5746994
3112208	Capela Nova	MG	0.0	0.00	-20.9185009	-43.6094017
3502903	Araçoiaba da Serra	SP	0.0	0.00	-23.54400063	-47.65169907
3112307	Capelinha	MG	0.0	0.00	-17.69370079	-42.49219894
3503000	Aramina	SP	0.0	0.00	-20.14509964	-47.82089996
3112406	Capetinga	MG	0.0	0.00	-20.65859985	-47.03250122
3503109	Arandu	SP	0.0	0.00	-23.17770004	-4.906399918
3112505	Capim Branco	MG	0.0	0.00	-19.57119942	-44.1692009
3112604	Capinópolis	MG	0.0	0.00	-18.68829918	-49.58259964
3503158	Arapeí	SP	0.0	0.00	-22.67320061	-44.43870163
3503208	Araraquara	SP	0.0	0.00	-21.79120064	-48.18180084
3112653	Capitão Andrade	MG	0.0	0.00	-19.05200005	-41.82249832
3503307	Araras	SP	0.0	0.00	-22.34639931	-47.32429886
3112703	Capitão Enéas	MG	0.0	0.00	-16.1364994	-43.66519928
3503356	Arco-Íris	SP	0.0	0.00	-21.76639938	-5.043109894
3112802	Capitólio	MG	0.0	0.00	-20.61339951	-46.14490128
3503406	Arealva	SP	0.0	0.00	-22.07620049	-48.98120117
3112901	Caputira	MG	1.0	1.11	-20.17679977	-42.25310135
3503505	Areias	SP	0.0	0.00	-22.67340088	-4.471260071
3113008	Caraí	MG	1.0	4.48	-17.17449951	-41.58449936
3503604	Areiópolis	SP	0.0	0.00	-22.6317997	-48.65309906

3113107	Caranaíba	MG	0.0	0.00	-20.89209938	-43.72230148
3503703	Ariranha	SP	0.0	0.00	-21.18569946	-4.877949905
3113206	Carandaí	MG	0.0	0.00	-20.99740028	-43.84560013
3503802	Artur Nogueira	SP	0.0	0.00	-22.56159973	-47.13270187
3113305	Carangola	MG	2.0	6.19	-20.70359993	-42.08860016
3503901	Arujá	SP	0.0	0.00	-2.338610077	-46.31919861
3113404	Caratinga	MG	56.0	6.57	-1.971260071	-42.10300064
3503950	Aspásia	SP	0.0	0.00	-20.18379974	-50.72880173
3113503	Carbonita	MG	0.0	0.00	-17.49539948	-43.05870056
3504008	Assis	SP	0.0	0.00	-2.260160065	-50.42110062
3113602	Careaçu	MG	0.0	0.00	-0.220797005	-45.66540146
3504107	Atibaia	SP	1.0	0.00	-23.12369919	-46.58739853
3113701	Carlos Chagas	MG	0.0	0.00	-17.67490005	-40.84220123
3504206	Auriflama	SP	0.0	0.00	-20.64450073	-50.5802002
3113800	Carmésia	MG	0.0	0.00	-19.06110001	-43.17570114
3504305	Avaí	SP	0.0	0.00	-22.1868	-49.31650162
3113909	Carmo da Cachoeira	MG	0.0	0.00	-21.45499992	-45.19739914
3504404	Avanhandava	SP	0.0	0.00	-21.46039963	-49.94810104
3114006	Carmo da Mata	MG	0.0	0.00	-20.56080055	-44.89260101
3504503	Avaré	SP	0.0	0.00	-23.07460022	-4.889519882
3114105	Carmo de Minas	MG	0.0	0.00	-22.09289932	-45.16210175
3504602	Bady Bassitt	SP	0.0	0.00	-20.93149948	-49.4382019
3114204	Carmo do Cajuru	MG	0.0	0.00	-20.19739914	-44.70190048
3504701	Balbinos	SP	0.0	0.00	-21.8932991	-49.33399963
3114303	Carmo do Paranaíba	MG	1.0	3.36	-1.890870094	-46.18569946
3504800	Bálsamo	SP	0.0	0.00	-20.70960045	-49.55210114
3114402	Carmo do Rio Claro	MG	0.0	0.00	-20.97389984	-46.09960175
3114501	Carmópolis de Minas	MG	0.0	0.00	-20.56290054	-44.64889908
3504909	Bananal	SP	0.0	0.00	-22.73390007	-44.33480072
3114550	Carneirinho	MG	0.0	0.00	-19.75090027	-50.8302002
3505005	Barão de Antonina	SP	0.0	0.00	-23.58340073	-4.957210159
3505104	Barbosa	SP	0.0	0.00	-21.29080009	-49.92240143
3114600	Carrancas	MG	0.0	0.00	-21.49740028	-44.61320114
3505203	Bariri	SP	0.0	0.00	-22.06649971	-48.71879959
3114808	Carvalhos	MG	0.0	0.00	-22.0237999	-44.48939896
3505302	Barra Bonita	SP	0.0	0.00	-22.47949982	-48.54169846
3114907	Casa Grande	MG	0.0	0.00	-20.83679962	-43.93909836
3505351	Barra do Chapéu	SP	0.0	0.00	-24.4442997	-49.08549881
3115003	Cascalho Rico	MG	0.0	0.00	-18.56949997	-47.87760162
3505401	Barra do Turvo	SP	0.0	0.00	-24.88870049	-48.42760086
3115201	Conceição da Barra de Minas	MG	0.0	0.00	-21.14649963	-44.49440002
3505500	Barretos	SP	0.0	0.00	-20.51429939	-48.65250015
3115300	Cataguases	MG	0.0	0.00	-21.34309959	-42.67359924
3505609	Barrinha	SP	0.0	0.00	-21.23250008	-48.09569931
3115359	Catas Altas	MG	0.0	0.00	-20.07600021	-43.40890121
3505708	Barueri	SP	0.0	0.00	-23.50559998	-4.687670136
3115409	Catas Altas da Noruega	MG	0.0	0.00	-2.067099953	-43.50130081
3505807	Bastos	SP	0.0	0.00	-21.9503994	-50.74459839
3115458	Catuji	MG	0.0	0.00	-17.37249947	-41.49900055
3505906	Batatais	SP	0.0	0.00	-20.86779976	-47.57509995
3115474	Catuti	MG	0.0	0.00	-15.33650017	-43.09870148
3506003	Bauru	SP	0.0	0.00	-22.25419998	-49.12689972
3115508	Caxambu	MG	0.0	0.00	-21.97879982	-44.94850159
3506102	Bebedouro	SP	0.0	0.00	-20.9409008	-48.51089859
3115607	Cedro do Abaeté	MG	0.0	0.00	-19.12229919	-45.69950104
3506201	Bento de Abreu	SP	0.0	0.00	-21.33189964	-50.86249924
3115706	Central de Minas	MG	0.0	0.00	-18.76950073	-4.129550171
3506300	Bernardino de Campos	SP	0.0	0.00	-23.03070068	-49.49200058
3115805	Centralina	MG	0.0	0.00	-18.63369942	-49.1631012
3115904	Chácara	MG	0.0	0.00	-21.68759918	-43.21429825
3506409	Bilac	SP	0.0	0.00	-21.42760086	-50.47969818
3116001	Chalé	MG	2.0	3.54	-20.03190041	-41.66130066
3506508	Birigui	SP	0.0	0.00	-21.26210022	-5.035139847
3116100	Chapada do Norte	MG	0.0	0.00	-17.14819908	-42.41260147
3506607	Biritiba-Mirim	SP	0.0	0.00	-23.62459946	-46.02209854
3116159	Chapada Gaúcha	MG	0.0	0.00	-1.547000027	-45.45650101
3506706	Boa Esperança do Sul	SP	0.0	0.00	-21.93519974	-48.46390152
3116209	Chiador	MG	0.0	0.00	-2.200390053	-43.0304985
3506805	Bocaina	SP	0.0	0.00	-22.10540009	-48.53010178
3116308	Cipotânea	MG	0.0	0.00	-20.92480087	-43.35879898
3506904	Bofete	SP	0.0	0.00	-23.12899971	-48.28649902
3116407	Claraval	MG	0.0	0.00	-20.36260033	-47.24160004
3507001	Boituva	SP	0.0	0.00	-23.28790092	-47.67490005
3116506	Claro dos Poções	MG	0.0	0.00	-17.07099915	-44.23070145
3507100	Bom Jesus dos Perdões	SP	0.0	0.00	-2.317270088	-46.47919846
3116605	Cláudio	MG	0.0	0.00	-20.39830017	-44.77700043
3507159	Bom Sucesso de Itararé	SP	0.0	0.00	-24.31609917	-49.16469955
3116704	Coimbra	MG	0.0	0.00	-20.84420013	-4.279230118
3507209	Borá	SP	0.0	0.00	-22.24440002	-50.50230026
3116803	Coluna	MG	1.0	1.11	-18.24860001	-42.84069824
3116902	Comendador Gomes	MG	0.0	0.00	-19.67329979	-49.07130051
3507308	Boracéia	SP	0.0	0.00	-22.1704998	-48.78789902
3507407	Borborema	SP	0.0	0.00	-21.61090088	-49.07239914
3117009	Comercinho	MG	1.0	1.21	-16.28689957	-41.76900101
3507456	Borebi	SP	0.0	0.00	-22.68020058	-4.899240112
3117108	Conceição da Aparecida	MG	0.0	0.00	-21.09889984	-4.622639847
3507506	Botucatu	SP	0.0	0.00	-22.86310005	-48.46889877

3117207	Conceição das Pedras	MG	0.0	0.00	-22.14819908	-45.42599869
3117306	Conceição das Alagoas	MG	0.0	0.00	-19.95980072	-48.36169815
3507605	Bragança Paulista	SP	0.0	0.00	-22.93869972	-46.55619812
3507704	Braúna	SP	0.0	0.00	-21.55330086	-50.33980179
3117405	Conceição de Ipanema	MG	1.0	2.24	-19.91419983	-41.68780136
3507753	Brejo Alegre	SP	0.0	0.00	-21.17700005	-50.21030045
3117504	Conceição do Mato Dentro	MG	0.0	0.00	-18.94260025	-43.50559998
3507803	Brodowski	SP	0.0	0.00	-21.04969978	-47.62860107
3117603	Conceição do Pará	MG	0.0	0.00	-19.78619957	-44.87239838
3507902	Brotas	SP	0.0	0.00	-22.27330017	-48.08190155
3117702	Conceição do Rio Verde	MG	0.0	0.00	-21.89789963	-45.09189987
3508009	Buri	SP	0.0	0.00	-2.375219917	-48.57580185
3117801	Conceição dos Ouros	MG	0.0	0.00	-22.44949913	-45.77740097
3508108	Buritama	SP	0.0	0.00	-21.04929924	-50.20130157
3117836	Cônego Marinho	MG	0.0	0.00	-14.97570038	-44.60279846
3508207	Buritizal	SP	0.0	0.00	-20.20960045	-47.69309998
3117900	Congonhal	MG	0.0	0.00	-22.14450073	-46.04240036
3508306	Cabrália Paulista	SP	0.0	0.00	-22.48929977	-4.937329865
3118007	Congonhas	MG	0.0	0.00	-20.51689911	-43.86209869
3508405	Cabreúva	SP	0.0	0.00	-23.30130005	-47.08060074
3118106	Congonhas do Norte	MG	0.0	0.00	-18.84900093	-43.69620132
3118205	Conquista	MG	0.0	0.00	-19.88430023	-47.62490082
3508504	Caçapava	SP	0.0	0.00	-23.10390091	-45.71390152
3508603	Cachoeira Paulista	SP	0.0	0.00	-22.70249939	-4.499209976
3118304	Conselheiro Lafaiete	MG	0.0	0.00	-20.66699982	-43.78979874
3508702	Caconde	SP	0.0	0.00	-21.54000092	-46.61750031
3118403	Conselheiro Pena	MG	2.0	8.99	-19.16110039	-41.44689941
3508801	Cafelândia	SP	0.0	0.00	-21.73119926	-49.5489006
3118502	Consolação	MG	0.0	0.00	-2.253910065	-4.5917099
3508900	Caiabu	SP	0.0	0.00	-21.95050049	-51.22409821
3118601	Contagem	MG	2.0	0.00	-19.88780022	-44.08480072
3509007	Caieiras	SP	0.0	0.00	-23.37680054	-46.74520111
3118700	Coqueiral	MG	0.0	0.00	-21.17000008	-45.41630173
3509106	Caiuá	SP	0.0	0.00	-21.79980087	-51.98410034
3118809	Coração de Jesus	MG	0.0	0.00	-16.6147995	-44.37950134
3509205	Cajamar	SP	0.0	0.00	-23.35079956	-46.87229919
3118908	Cordisburgo	MG	0.0	0.00	-1.909300041	-44.20650101
3509254	Cajati	SP	0.0	0.00	-24.77230072	-48.19520187
3119005	Cordislândia	MG	0.0	0.00	-2.178300095	-45.66270065
3509304	Cajobi	SP	0.0	0.00	-20.87409973	-48.83959961
3119104	Corinto	MG	0.0	0.00	-18.33670044	-44.61849976
3509403	Cajuru	SP	0.0	0.00	-21.27619934	-47.3105011
3119203	Coroaci	MG	0.0	0.00	-18.61759949	-42.26959991
3509452	Campina do Monte Alegre	SP	0.0	0.00	-23.60689926	-48.44290161
3119302	Coromandel	MG	0.0	0.00	-18.4137001	-4.713729858
3509502	Campinas	SP	1.0	0.00	-22.88430023	-47.04449844
3119401	Coronel Fabriciano	MG	0.0	0.00	-19.45879936	-42.69049835
3509601	Campo Limpo Paulista	SP	0.0	0.00	-23.21839905	-46.76039886
3119500	Coronel Murta	MG	0.0	0.00	-1.659199905	-42.17699814
3509700	Campos do Jordão	SP	0.0	0.00	-22.70199966	-45.53350067
3119609	Coronel Pacheco	MG	0.0	0.00	-21.60569954	-43.29370117
3509809	Campos Novos Paulista	SP	0.0	0.00	-22.61090088	-50.01010132
3119708	Coronel Xavier Chaves	MG	0.0	0.00	-21.02569962	-44.19749832
3509908	Cananéia	SP	0.0	0.00	-25.01930046	-48.00899887
3119807	Córrego Danta	MG	0.0	0.00	-19.78440094	-45.98160172
3119906	Córrego do Bom Jesus	MG	0.0	0.00	-22.6352005	-45.9939003
3509957	Canas	SP	0.0	0.00	-22.72979927	-45.04190063
3510005	Cândido Mota	SP	0.0	0.00	-22.80900002	-50.42250061
3119955	Córrego Fundo	MG	0.0	0.00	-20.44989967	-45.53409958
3510104	Cândido Rodrigues	SP	0.0	0.00	-21.34269905	-48.62889862
3120003	Córrego Novo	MG	0.0	0.00	-19.83069992	-42.44820023
3510153	Canitar	SP	0.0	0.00	-23.0189991	-49.79199982
3120102	Couto de Magalhães de Minas	MG	0.0	0.00	-18.08499908	-43.4408989
3510203	Capão Bonito	SP	0.0	0.00	-24.03930092	-48.29040146
3120151	Crisólita	MG	0.0	0.00	-17.24110031	-40.96149826
3510302	Capela do Alto	SP	0.0	0.00	-23.46719933	-4.773130035
3120201	Cristais	MG	0.0	0.00	-20.81229973	-45.55810165
3510401	Capivari	SP	0.0	0.00	-22.98110008	-47.47850037
3120300	Cristália	MG	0.0	0.00	-16.71969986	-42.82139969
3120409	Cristiano Otoni	MG	0.0	0.00	-20.83609962	-43.8280983
3510609	Carapicuíba	SP	0.0	0.00	-23.54949951	-46.84270096
3120508	Cristina	MG	0.0	0.00	-22.2064991	-45.2901001
3510708	Cardoso	SP	0.0	0.00	-20.06800079	-49.94800186
3120607	Crucilândia	MG	0.0	0.00	-20.40539932	-0.044360699
3510807	Casa Branca	SP	0.0	0.00	-21.80080032	-47.0882988
3120706	Cruzeiro da Fortaleza	MG	0.0	0.00	-18.97260094	-46.67710114
3510906	Cássia dos Coqueiros	SP	0.0	0.00	-21.26350021	-47.14450073
3120805	Cruzília	MG	0.0	0.00	-21.73180008	-44.79899979
3511003	Castilho	SP	0.0	0.00	-20.89900017	-51.57049942
3120839	Cuparaque	MG	0.0	0.00	-19.00889969	-41.12960052
3511102	Catanduva	SP	0.0	0.00	-21.13380051	-48.96469879
3120870	Curral de Dentro	MG	0.0	0.00	-15.86320019	-41.7663002
3511201	Catiguá	SP	0.0	0.00	-21.06270027	-49.0530014
3120904	Curvelo	MG	0.0	0.00	-18.82119942	-44.44919968
3511300	Cedral	SP	0.0	0.00	-20.91320038	-49.26340103
3121001	Datas	MG	0.0	0.00	-18.47130013	-43.64870071
3511409	Cerqueira César	SP	0.0	0.00	-2.306030083	-49.14450073

3121100	Delfim Moreira	MG	0.0	0.00	-22.50760078	-45.2887001
3511508	Cerquilho	SP	0.0	0.00	-23.18740082	-47.75719833
3121209	Delfinópolis	MG	0.0	0.00	-20.35750008	-46.77420044
3511607	Cesário Lange	SP	0.0	0.00	-23.21680069	-47.90420151
3121258	Delta	MG	0.0	0.00	-19.93639946	-47.81110001
3511706	Charqueada	SP	0.0	0.00	-22.53009987	-47.74700165
3121308	Descoberto	MG	0.0	0.00	-21.44779968	-42.96469879
3511904	Clementina	SP	0.0	0.00	-21.57060051	-50.46049881
3121407	Desterro de Entre Rios	MG	0.0	0.00	-20.63059998	-44.27399826
3512001	Colina	SP	0.0	0.00	-20.7465992	-4.858909988
3121506	Desterro do Melo	MG	0.0	0.00	-2.115159988	-43.52080154
3512100	Colômbia	SP	0.0	0.00	-20.26709938	-48.72159958
3121605	Diamantina	MG	0.0	0.00	-17.99329948	-0.043610699
3512209	Conchal	SP	0.0	0.00	-2.23696003	-47.1427002
3121704	Diogo de Vasconcelos	MG	0.0	0.00	-20.4822998	-43.18460083
3512308	Conchas	SP	0.0	0.00	-22.96549988	-48.04729843
3121803	Dionísio	MG	0.0	0.00	-19.83810043	-42.67910004
3512407	Cordeirópolis	SP	0.0	0.00	-22.47960091	-47.41490173
3121902	Divinésia	MG	0.0	0.00	-20.9890995	-4.299850082
3512506	Coroados	SP	0.0	0.00	-21.37579918	-50.30390167
3122009	Divino	MG	0.0	0.00	-20.59079933	-42.17630005
3512605	Coronel Macedo	SP	0.0	0.00	-23.63010025	-49.30709839
3122108	Divino das Laranjeiras	MG	0.0	0.00	-18.71310043	-41.50749969
3512704	Corumbataí	SP	0.0	0.00	-22.2364006	-47.61130142
3122207	Divinolândia de Minas	MG	0.0	0.00	-18.78339958	-42.57569885
3512803	Cosmópolis	SP	0.0	0.00	-22.65189934	-47.18659973
3122306	Divinópolis	MG	0.0	0.00	-20.12470055	-44.93149948
3512902	Cosmorama	SP	0.0	0.00	-2.04428997	-4.977510071
3122355	Divisa Alegre	MG	0.0	0.00	-15.69820023	-41.37450027
3513009	Cotia	SP	0.0	0.00	-23.67510033	-4.696089935
3122405	Divisa Nova	MG	0.0	0.00	-21.5189991	-46.2466011
3513108	Cravinhos	SP	0.0	0.00	-21.34009933	-47.74489975
3122454	Divisópolis	MG	0.0	0.00	-15.76290035	-40.92869949
3513207	Cristais Paulista	SP	0.0	0.00	-20.37120056	-47.40119934
3122470	Dom Bosco	MG	0.0	0.00	-16.73999977	-46.29040146
3513306	Cruzália	SP	0.0	0.00	-22.7378006	-50.77000046
3122504	Dom Cavati	MG	0.0	0.00	-0.193922005	-42.09609985
3513405	Cruzeiro	SP	0.0	0.00	-22.55179977	-45.00630188
3122603	Dom Joaquim	MG	0.0	0.00	-18.93120003	-4.326110077
3513504	Cubatão	SP	0.0	0.00	-23.86560059	-46.40859985
3122702	Dom Silvério	MG	0.0	0.00	-20.13549995	-42.94749832
3122801	Dom Viçoso	MG	0.0	0.00	-22.22909927	-45.14279938
3513603	Cunha	SP	0.0	0.00	-23.05310059	-4.494240189
3513702	Descalvado	SP	0.0	0.00	-21.88139915	-47.65459824
3122900	Dona Eusébia	MG	0.0	0.00	-21.32189941	-42.80189896
3513801	Diadema	SP	0.0	0.00	-23.69729996	-46.61169815
3123007	Dores de Campos	MG	0.0	0.00	-21.10149956	-43.9917984
3513850	Dirce Reis	SP	0.0	0.00	-20.44890022	-50.62850189
3123106	Dores de Guanhães	MG	0.0	0.00	-19.05450058	-42.93249893
3513900	Divinolândia	SP	0.0	0.00	-21.66180038	-46.69789886
3123205	Dores do Indaiá	MG	0.0	0.00	-19.49060059	-45.54840088
3514007	Dobrada	SP	0.0	0.00	-21.51619911	-48.35789871
3123304	Dores do Turvo	MG	0.0	0.00	-21.02540016	-43.16830063
3514106	Dois Córregos	SP	0.0	0.00	-22.39620018	-48.3409996
3123403	Doresópolis	MG	0.0	0.00	-20.29260063	-45.8885994
3514205	Dolcinópolis	SP	0.0	0.00	-20.11680031	-50.5284996
3123502	Douradoquara	MG	0.0	0.00	-18.43810081	-47.60570145
3514304	Dourado	SP	0.0	0.00	-22.11739922	-48.33380127
3123528	Durandé	MG	5.0	6.74	-20.14859962	-41.79669952
3514403	Dracena	SP	0.0	0.00	-21.55719948	-51.58449936
3123601	Elói Mendes	MG	0.0	0.00	-21.61330032	-45.59820175
3514502	Duartina	SP	0.0	0.00	-22.3932991	-49.41360092
3123700	Engenheiro Caldas	MG	0.0	0.00	-19.1291008	-42.01979828
3514601	Dumont	SP	0.0	0.00	-2.124780083	-47.98040009
3123809	Engenheiro Navarro	MG	0.0	0.00	-17.29409981	-4.401869965
3514700	Echaporã	SP	0.0	0.00	-22.4484005	-50.20080185
3123858	Entre Folhas	MG	7.0	1.35	-19.65640068	-42.24250031
3514809	Eldorado	SP	0.0	0.00	-24.49539948	-48.2336998
3123908	Entre Rios de Minas	MG	0.0	0.00	-20.70429993	-44.09790039
3514908	Elias Fausto	SP	0.0	0.00	-23.07089996	-47.37080002
3124005	Ervália	MG	0.0	0.00	-20.84950066	-42.60169983
3514924	Elisiário	SP	0.0	0.00	-21.16040039	-4.909230042
3124104	Esmeraldas	MG	1.0	1.66	-19.7336998	-44.30860138
3514957	Embaúba	SP	0.0	0.00	-2.095299912	-48.8484993
3124203	Espera Feliz	MG	0.0	0.00	-20.59670067	-41.92869949
3515004	Embu	SP	0.0	0.00	-23.6515007	-46.8526001
3124302	Espinosa	MG	0.0	0.00	-14.8597002	-42.96659851
3515103	Embu-Guaçu	SP	0.0	0.00	-23.85460091	-46.83110046
3124401	Espírito Santo do Dourado	MG	0.0	0.00	-22.00970078	-45.98759842
3515129	Emilianópolis	SP	0.0	0.00	-21.80150032	-51.47259903
3124500	Estiva	MG	0.0	0.00	-22.45980072	-46.01459885
3515152	Engenheiro Coelho	SP	0.0	0.00	-22.48769951	-47.1753006
3124609	Estrela Dalva	MG	0.0	0.00	-21.70759964	-42.46780014
3515186	Espírito Santo do Pinhal	SP	0.0	0.00	-22.19260025	-4.679380035
3124708	Estrela do Indaiá	MG	0.0	0.00	-19.59250069	-45.80920029
3515194	Espírito Santo do Turvo	SP	0.0	0.00	-22.66990089	-49.43299866
3124807	Estrela do Sul	MG	0.0	0.00	-18.71610069	-47.72719955

3515202	Estrela d'Oeste	SP	0.0	0.00	-20.26609993	-50.41180038
3124906	Eugenópolis	MG	0.0	0.00	-2.101479912	-42.22480011
3515301	Estrela do Norte	SP	0.0	0.00	-22.48509979	-51.66970062
3125002	Ewbank da Câmara	MG	0.0	0.00	-21.57570076	-43.56110001
3515350	Euclides da Cunha Paulista	SP	0.0	0.00	-22.51959991	-52.58990097
3125101	Extrema	MG	0.0	0.00	-2.282579994	-4.629059982
3515400	Fartura	SP	0.0	0.00	-23.38990021	-49.52000046
3125200	Fama	MG	0.0	0.00	-21.46619987	-45.81539917
3515509	Fernandópolis	SP	0.0	0.00	-2.027260017	-50.28350067
3125309	Faria Lemos	MG	0.0	0.00	-20.78219986	-42.03639984
3515608	Fernando Prestes	SP	0.0	0.00	-21.31089973	-48.69589996
3125408	Felício dos Santos	MG	1.0	1.94	-18.13310051	-43.24029922
3515657	Fernão	SP	0.0	0.00	-22.36590004	-49.54249954
3125507	São Gonçalo do Rio Preto	MG	0.0	0.00	-18.07690048	-43.35960007
3515707	Ferraz de Vasconcelos	SP	0.0	0.00	-23.56139946	-46.37419891
3125606	Felisburgo	MG	0.0	0.00	-16.63809967	-40.73379898
3515806	Flora Rica	SP	0.0	0.00	-2.169930077	-51.37469864
3125705	Felixlândia	MG	0.0	0.00	-18.70389938	-44.95980072
3515905	Floreal	SP	0.0	0.00	-20.6704998	-50.1556015
3125804	Fernandes Tourinho	MG	0.0	0.00	-19.10230064	-42.09719849
3516002	Flórida Paulista	SP	0.0	0.00	-21.54080009	-51.17110062
3125903	Ferros	MG	0.0	0.00	-1.924609947	-42.94120026
3516101	Florínia	SP	0.0	0.00	-22.88120079	-50.69580078
3125952	Fervedouro	MG	0.0	0.00	-20.68129921	-42.34769821
3516200	Franca	SP	0.0	0.00	-20.55520058	-47.38069916
3126000	Florestal	MG	0.0	0.00	-1.98696003	-44.44329834
3516309	Francisco Morato	SP	0.0	0.00	-23.27449989	-46.72399902
3126109	Formiga	MG	0.0	0.00	-20.55509949	-45.51779938
3516408	Franco da Rocha	SP	0.0	0.00	-2.331469917	-46.73590088
3126208	Formoso	MG	0.0	0.00	-15.13029957	-46.15909958
3516507	Gabriel Monteiro	SP	0.0	0.00	-21.49950027	-50.5646019
3126307	Fortaleza de Minas	MG	0.0	0.00	-20.88909912	-46.77230072
3516606	Gália	SP	0.0	0.00	-22.32029915	-49.57780075
3126406	Fortuna de Minas	MG	1.0	3.70	-19.55850029	-44.50519943
3516705	Garça	SP	0.0	0.00	-22.23089981	-49.69010162
3126505	Francisco Badaró	MG	0.0	0.00	-16.97839928	-42.31019974
3516804	Gastão Vidigal	SP	0.0	0.00	-20.80489922	-50.20339966
3126604	Francisco Dumont	MG	0.0	0.00	-17.47459984	-4.427360153
3516853	Gavião Peixoto	SP	0.0	0.00	-21.79159927	-48.44810104
3126703	Francisco Sá	MG	0.0	0.00	-16.39480019	-43.47999954
3516903	General Salgado	SP	0.0	0.00	-20.63879967	-50.41619873
3126752	Franciscópolis	MG	2.0	3.45	-17.99399948	-41.96829987
3517000	Getulina	SP	0.0	0.00	-21.78359985	-5.002830124
3126802	Frei Gaspar	MG	6.0	1.02	-1.814049912	-41.51119995
3517109	Glicério	SP	0.0	0.00	-21.33989906	-50.19910049
3126901	Frei Inocêncio	MG	0.0	0.00	-18.52050018	-41.88050079
3517208	Guaiçara	SP	0.0	0.00	-21.57299995	-49.76409912
3126950	Frei Lagonegro	MG	0.0	0.00	-18.14439964	-42.75690079
3517307	Guaimbê	SP	0.0	0.00	-2.187890053	-49.86109924
3127008	Fronteira	MG	0.0	0.00	-20.22929955	-49.15930176
3517406	Guaíra	SP	0.0	0.00	-20.31430054	-48.36410141
3127057	Fronteira dos Vales	MG	0.0	0.00	-16.89640045	-40.83530045
3517505	Guapiaçu	SP	0.0	0.00	-20.74379921	-49.1908989
3127073	Fruta de Leite	MG	0.0	0.00	-16.12630081	-42.52090073
3127107	Frutal	MG	0.0	0.00	-20.03610039	-49.00059891
3517604	Guapiara	SP	0.0	0.00	-24.21279907	-48.55590057
3517703	Guará	SP	0.0	0.00	-20.48240089	-47.77590179
3127206	Funilândia	MG	0.0	0.00	-19.36190033	-44.07529831
3517802	Guaraçaí	SP	0.0	0.00	-21.08740044	-51.2804985
3127305	Galiléia	MG	0.0	0.00	-18.88159943	-41.54610062
3517901	Guaraci	SP	0.0	0.00	-20.37520027	-49.00289917
3127339	Gameleiras	MG	0.0	0.00	-14.97249985	-43.25989914
3518008	Guarani d'Oeste	SP	0.0	0.00	-20.06739998	-50.34540176
3127354	Glaucilândia	MG	0.0	0.00	-16.90460014	-43.6405983
3518107	Guarantã	SP	0.0	0.00	-21.91629982	-49.58879852
3127370	Goiabeira	MG	0.0	0.00	-19.0223999	-41.23590088
3518206	Guararapes	SP	0.0	0.00	-2.128520012	-50.69589996
3127388	Goianá	MG	0.0	0.00	-21.55620003	-43.19010162
3518305	Guararema	SP	0.0	0.00	-23.42779922	-46.05870056
3127404	Gonçalves	MG	0.0	0.00	-22.67399979	-45.82600021
3518404	Guaratinguetá	SP	0.0	0.00	-2.279380035	-45.23320007
3127503	Gonzaga	MG	0.0	0.00	-18.87400055	-42.5060997
3518503	Guareí	SP	0.0	0.00	-2.337030029	-48.21960068
3127602	Gouveia	MG	0.0	0.00	-18.5223999	-43.83280182
3518602	Guariba	SP	0.0	0.00	-21.39609909	-48.22669983
3127701	Governador Valadares	MG	0.0	0.00	-18.7840004	-41.96419907
3518701	Guarujá	SP	0.0	0.00	-23.95190048	-46.23619843
3127800	Grão Mogol	MG	0.0	0.00	-16.46450043	-42.99409866
3127909	Grupiara	MG	0.0	0.00	-1.848889923	-47.76179886
3518800	Guarulhos	SP	0.0	0.00	-23.40259933	-46.45510101
3518859	Guatapará	SP	0.0	0.00	-21.45359993	-47.98020172
3128006	Guanhães	MG	0.0	0.00	-1.883440018	-42.85369873
3518909	Guzolândia	SP	0.0	0.00	-20.62899971	-50.71379852
3128105	Guapé	MG	0.0	0.00	-20.76539993	-4.592200089
3519006	Herculândia	SP	0.0	0.00	-21.96139908	-5.037329865
3128204	Guaraciaba	MG	0.0	0.00	-20.55610085	-43.02149963
3519055	Holambra	SP	0.0	0.00	-22.62960052	-47.06520081

3128253	Guaraciama	MG	1.0	2.12	-17.0795002	-43.62120056
3519071	Hortolândia	SP	0.0	0.00	-22.8784008	-47.20880127
3128303	Guaranésia	MG	0.0	0.00	-21.27799988	-46.80810165
3519105	Iacanga	SP	0.0	0.00	-21.89109993	-49.04000092
3128402	Guarani	MG	0.0	0.00	-21.35829926	-43.05879974
3519204	Iacri	SP	0.0	0.00	-21.80019951	-50.61970139
3128501	Guarará	MG	0.0	0.00	-21.75600052	-43.02330017
3519253	Iaras	SP	0.0	0.00	-22.81909943	-49.10749817
3128600	Guarda-Mor	MG	0.0	0.00	-17.73360062	-47.1269989
3519303	Ibaté	SP	0.0	0.00	-2.195079994	-48.0279007
3128709	Guaxupé	MG	0.0	0.00	-21.29140091	-46.6841011
3519402	Ibirá	SP	0.0	0.00	-21.0739994	-49.22100067
3128808	Guidoval	MG	0.0	0.00	-21.1678009	-42.7867012
3519501	Ibirarema	SP	0.0	0.00	-22.81360054	-50.07709885
3128907	Guimarânia	MG	0.0	0.00	-18.81629944	-46.75479889
3519600	Ibitinga	SP	0.0	0.00	-21.79319954	-48.84859848
3129004	Guiricema	MG	0.0	0.00	-21.01580048	-42.70080185
3129103	Gurinhatã	MG	0.0	0.00	-19.09860039	-49.86959839
3519709	Ibiúna	SP	0.0	0.00	-23.80279922	-4.721580124
3519808	Icém	SP	0.0	0.00	-20.36689949	-49.18640137
3129202	Heliodora	MG	0.0	0.00	-22.05019951	-45.54169846
3519907	Iepê	SP	0.0	0.00	-22.64850044	-51.05739975
3129301	Iapu	MG	0.0	0.00	-19.3477993	-4.224359894
3520004	Igaraçu do Tietê	SP	0.0	0.00	-22.54179955	-48.5746994
3129400	Ibertioga	MG	0.0	0.00	-21.45100021	-4.3957901
3520103	Igarapava	SP	0.0	0.00	-20.06419945	-47.68270111
3129509	Ibiá	MG	0.0	0.00	-19.55890083	-46.59600067
3520202	Igaratá	SP	0.0	0.00	-23.1352005	-46.1507988
3129608	Ibiaí	MG	0.0	0.00	-16.82929993	-44.79119873
3520301	Iguape	SP	0.0	0.00	-24.54969978	-47.47510147
3129657	Ibiracatu	MG	0.0	0.00	-15.69649982	-44.14550018
3115102	Cássia	MG	0.0	0.00	-20.54879951	-46.92770004
3520426	Ilha Comprida	SP	0.0	0.00	-24.85989952	-47.71680069
3129806	Ibirité	MG	0.0	0.00	-0.200240994	-44.06900024
3520442	Ilha Solteira	SP	0.0	0.00	-20.43110085	-5.125759888
3129905	Ibitiúra de Minas	MG	0.0	0.00	-22.06819916	-46.40869904
3520509	Indaiatuba	SP	0.0	0.00	-23.10639954	-47.20270157
3130002	Ibituruna	MG	0.0	0.00	-21.16799927	-44.78110123
3520608	Indiana	SP	0.0	0.00	-22.13439941	-51.2621994
3130051	Icaraí de Minas	MG	0.0	0.00	-16.22629929	-4.4917099
3520707	Indiaporã	SP	0.0	0.00	-19.94849968	-50.2641983
3130101	Igarapé	MG	0.0	0.00	-20.05949974	-44.32229996
3520806	Inúbia Paulista	SP	0.0	0.00	-21.73950005	-5.0957901
3130200	Igaratinga	MG	0.0	0.00	-19.95199966	-44.7193985
3520905	Ipaussu	SP	0.0	0.00	-23.06500053	-49.60839844
3130309	Iguatama	MG	0.0	0.00	-2.015699959	-45.75360107
3521002	Iperó	SP	0.0	0.00	-23.40439987	-47.62960052
3130408	Ijaci	MG	0.0	0.00	-21.17989922	-44.93460083
3521101	Ipeúna	SP	0.0	0.00	-22.42399979	-47.71620178
3130507	Ilicínea	MG	1.0	8.70	-20.93239975	-45.80239868
3521150	Ipiguá	SP	0.0	0.00	-2.064839935	-49.4034996
3130556	Imbé de Minas	MG	13.0	2.02	-19.62059975	-41.96839905
3521200	Iporanga	SP	0.0	0.00	-24.51269913	-48.54669952
3130606	Inconfidentes	MG	0.0	0.00	-22.33749962	-4.628269959
3521309	Ipuã	SP	0.0	0.00	-20.41729927	-48.06750107
3130655	Indaiabira	MG	1.0	1.36	-15.56280041	-42.14379883
3130705	Indianópolis	MG	0.0	0.00	-1.895299912	-47.85079956
3521408	Iracemápolis	SP	0.0	0.00	-22.59309959	-47.52460098
3521507	Irapuã	SP	0.0	0.00	-21.26790047	-49.39759827
3130804	Ingaí	MG	0.0	0.00	-2.14116993	-44.92599869
3521606	Irapuru	SP	0.0	0.00	-21.48609924	-51.34719849
3130903	Inhapim	MG	3.0	0.12	-19.49939919	-41.96620178
3521705	Itaberá	SP	0.0	0.00	-23.87439919	-49.14400101
3131000	Inhaúma	MG	0.0	0.00	-19.49880028	-44.41049957
3521804	Itaí	SP	0.0	0.00	-23.47559929	-49.05519867
3131109	Inimutaba	MG	0.0	0.00	-18.73019981	-44.29169846
3521903	Itajobi	SP	0.0	0.00	-21.35580063	-49.05419922
3131158	Ipaba	MG	0.0	0.00	-19.40950012	-42.37139893
3522000	Itaju	SP	0.0	0.00	-21.95140076	-48.79449844
3131208	Ipanema	MG	15.0	8.26	-19.75830078	-41.75009918
3522109	Itanhaém	SP	0.0	0.00	-24.09210014	-46.83530045
3131307	Ipatinga	MG	4.0	1.67	-19.43959999	-42.60079956
3131406	Ipiaçu	MG	0.0	0.00	-18.69910049	-4.993259811
3522158	Itaóca	SP	0.0	0.00	-24.61809921	-48.83919907
3522208	Itapecerica da Serra	SP	0.0	0.00	-23.73749924	-46.85919952
3131505	Ipuiúna	MG	0.0	0.00	-2.202879906	-46.13119888
3522307	Itapetininga	SP	0.0	0.00	-23.64550018	-48.12379837
3131604	Iraí de Minas	MG	0.0	0.00	-19.04470062	-47.45830154
3522406	Itapeva	SP	0.0	0.00	-23.91430092	-48.87210083
3131703	Itabira	MG	0.0	0.00	-19.60149956	-43.30120087
3522505	Itapevi	SP	0.0	0.00	-23.55509949	-46.97180176
3131802	Itabirinha de Mantena	MG	0.0	0.00	-18.54990005	-41.24470139
3522604	Itapira	SP	0.0	0.00	-22.43199921	-46.77230072
3131901	Itabirito	MG	0.0	0.00	-20.25449944	-43.81129837
3522653	Itapirapuã Paulista	SP	0.0	0.00	-24.5692997	-49.22330093
3132008	Itacambira	MG	0.0	0.00	-16.93610001	-0.433297005
3522703	Itápolis	SP	0.0	0.00	-21.54820061	-48.8158989

3132107	Itacarambi	MG	0.0	0.00	-15.18369961	-44.12360001
3522802	Itaporanga	SP	0.0	0.00	-23.67280006	-49.45899963
3132206	Itaguara	MG	0.0	0.00	-20.37260056	-4.453269959
3522901	Itapuí	SP	0.0	0.00	-22.25139999	-48.7016983
3132305	Itaipé	MG	2.0	1.70	-17.41060066	-41.65980148
3523008	Itapura	SP	0.0	0.00	-20.5970993	-51.44210052
3132404	Itajubá	MG	0.0	0.00	-22.42169952	-45.41540146
3523107	Itaquaquecetuba	SP	1.0	0.00	-23.46139908	-46.33449936
3132503	Itamarandiba	MG	0.0	0.00	-17.8572998	-42.87789917
3523206	Itararé	SP	0.0	0.00	-24.08110046	-49.30390167
3132602	Itamarati de Minas	MG	0.0	0.00	-2.141410065	-42.83729935
3523305	Itariri	SP	0.0	0.00	-24.29319954	-47.12850189
3132701	Itambacuri	MG	30.0	1.32	-18.15929985	-4.184669876
3523404	Itatiba	SP	1.0	0.00	-23.00670052	-46.8146019
3132800	Itambé do Mato Dentro	MG	0.0	0.00	-19.39710045	-43.35250092
3523503	Itatinga	SP	0.0	0.00	-23.1534996	-48.63000107
3132909	Itamogi	MG	0.0	0.00	-21.07659912	-47.05110168
3523602	Itirapina	SP	0.0	0.00	-22.29470062	-47.83449936
3133006	Itamonte	MG	0.0	0.00	-22.29019928	-44.75899887
3523701	Itirapuã	SP	0.0	0.00	-2.065800095	-47.16989899
3133105	Itanhandu	MG	0.0	0.00	-22.32080078	-44.92219925
3523800	Itobi	SP	0.0	0.00	-21.74900055	-46.92219925
3133204	Itanhomi	MG	3.0	2.53	-19.16119957	-41.82109833
3523909	Itu	SP	0.0	0.00	-23.30739975	-47.28509903
3133303	Itaobim	MG	0.0	0.00	-16.58399963	-41.53469849
3133402	Itapagipe	MG	0.0	0.00	-19.79570007	-49.4239006
3524006	Itupeva	SP	0.0	0.00	-23.14669991	-47.0685997
3524105	Ituverava	SP	0.0	0.00	-20.33040047	-47.80849838
3133501	Itapecerica	MG	0.0	0.00	-20.45969963	-45.10910034
3524204	Jaborandi	SP	0.0	0.00	-20.64970016	-48.41619873
3133600	Itapeva	MG	0.0	0.00	-22.7003994	-46.21030045
3524303	Jaboticabal	SP	0.0	0.00	-21.21850014	-48.28839874
3133709	Itatiaiuçu	MG	0.0	0.00	-2.021010017	-44.45410156
3133758	Itaú de Minas	MG	0.0	0.00	-20.73800087	-46.77249908
3524402	Jacareí	SP	0.0	0.00	-23.29859924	-45.99160004
3524501	Jaci	SP	0.0	0.00	-20.94589996	-49.58160019
3133808	Itaúna	MG	0.0	0.00	-20.07920074	-44.58969879
3524600	Jacupiranga	SP	0.0	0.00	-2.477659988	-48.05410004
3133907	Itaverava	MG	0.0	0.00	-20.68610001	-43.61449814
3524709	Jaguariúna	SP	0.0	0.00	-22.69239998	-47.01350021
3134004	Itinga	MG	0.0	0.00	-16.58790016	-4.183610153
3524808	Jales	SP	0.0	0.00	-20.28639984	-50.5542984
3134103	Itueta	MG	7.0	1.20	-19.37319946	-4.110480118
3134202	Ituiutaba	MG	0.0	0.00	-18.99300003	-49.54439926
3524907	Jambeiro	SP	0.0	0.00	-23.27910042	-45.71060181
3525003	Jandira	SP	0.0	0.00	-23.54439926	-46.90000153
3134301	Itumirim	MG	0.0	0.00	-21.29109955	-44.82529831
3134400	Iturama	MG	0.0	0.00	-19.7173996	-50.34540176
3525102	Jardinópolis	SP	0.0	0.00	-20.99220085	-47.82690048
3525201	Jarinu	SP	0.0	0.00	-23.11199951	-4.672169876
3134509	Itutinga	MG	0.0	0.00	-21.35219955	-44.71160126
3525300	Jaú	SP	0.0	0.00	-22.29570007	-48.55619812
3134608	Jaboticatubas	MG	0.0	0.00	-19.44149971	-0.437190018
3525409	Jeriquara	SP	0.0	0.00	-20.33989906	-47.57440186
3134707	Jacinto	MG	0.0	0.00	-1.618359947	-40.31470108
3134806	Jacuí	MG	0.0	0.00	-21.02569962	-0.046735699
3525508	Joanópolis	SP	0.0	0.00	-22.93939972	-46.20949936
3525607	João Ramalho	SP	0.0	0.00	-22.27770042	-50.79949951
3134905	Jacutinga	MG	0.0	0.00	-22.28849983	-46.60169983
3525706	José Bonifácio	SP	0.0	0.00	-2.108979988	-49.78039932
3135001	Jaguaraçu	MG	0.0	0.00	-1.963829994	-42.71879959
3525805	Júlio Mesquita	SP	0.0	0.00	-21.97319984	-49.79330063
3135050	Jaíba	MG	0.0	0.00	-15.24009991	-43.67100143
3525854	Jumirim	SP	0.0	0.00	-23.09980011	-47.78820038
3135076	Jampruca	MG	0.0	0.00	-18.46450043	-41.74160004
3525904	Jundiaí	SP	1.0	0.00	-23.19519997	-46.91329956
3135100	Janaúba	MG	0.0	0.00	-15.79769993	-43.39699936
3526001	Junqueirópolis	SP	0.0	0.00	-21.44989967	-51.4355011
3135209	Januária	MG	0.0	0.00	-15.3210001	-44.84909821
3526100	Juquiá	SP	0.0	0.00	-24.21419907	-47.65380096
3135308	Japaraíba	MG	0.0	0.00	-20.13369942	-45.5298996
3526209	Juquitiba	SP	0.0	0.00	-2.395549965	-47.02470016
3135357	Japonvar	MG	0.0	0.00	-15.94569969	-44.32960129
3526308	Lagoinha	SP	0.0	0.00	-23.08580017	-45.20709991
3135407	Jeceaba	MG	0.0	0.00	-20.55310059	-0.044048199
3526407	Laranjal Paulista	SP	0.0	0.00	-23.0121994	-47.86640167
3135456	Jenipapo de Minas	MG	0.0	0.00	-17.16959953	-42.21519852
3526506	Lavínia	SP	0.0	0.00	-21.15069962	-51.03329849
3135506	Jequeri	MG	0.0	0.00	-20.47450066	-42.62049866
3526605	Lavrinhas	SP	0.0	0.00	-22.52050018	-0.044889301
3135605	Jequitaí	MG	0.0	0.00	-17.21030045	-44.47710037
3526704	Leme	SP	0.0	0.00	-22.17239952	-47.33789825
3135704	Jequitibá	MG	0.0	0.00	-19.21220016	-44.00249863
3526803	Lençóis Paulista	SP	0.0	0.00	-22.67499924	-48.82559967
3135803	Jequitinhonha	MG	1.0	4.14	-16.39039993	-41.08309937
3526902	Limeira	SP	0.0	0.00	-22.59869957	-4.736389923
3135902	Jesuânia	MG	0.0	0.00	-22.0109005	-45.27610016

3527009	Lindóia	SP	0.0	0.00	-22.51339912	-46.65510178
3136009	Joaíma	MG	1.0	6.69	-16.77249908	-41.01570129
3527108	Lins	SP	0.0	0.00	-21.65019989	-49.68360138
3136108	Joanésia	MG	0.0	0.00	-19.21019936	-42.70539856
3527207	Lorena	SP	0.0	0.00	-22.79409981	-45.0603981
3136207	João Monlevade	MG	0.0	0.00	-19.8390007	-43.16210175
3527256	Lourdes	SP	0.0	0.00	-2.094930077	-5.023450089
3136306	João Pinheiro	MG	0.0	0.00	-17.60589981	-45.93500137
3527306	Louveira	SP	0.0	0.00	-23.08480072	-46.93759918
3136405	Joaquim Felício	MG	0.0	0.00	-17.67690086	-44.11190033
3527405	Lucélia	SP	0.0	0.00	-21.64450073	-50.99200058
3136504	Jordânia	MG	0.0	0.00	-15.88399982	-40.31430054
3527504	Lucianópolis	SP	0.0	0.00	-22.47360039	-49.55049896
3136520	José Gonçalves de Minas	MG	0.0	0.00	-16.9015007	-42.65679932
3527603	Luís Antônio	SP	0.0	0.00	-21.55290031	-47.78239822
3136553	José Raydan	MG	3.0	6.86	-18.24760056	-42.48099899
3527702	Luiziânia	SP	0.0	0.00	-2.167270088	-50.35100174
3136579	Josenópolis	MG	0.0	0.00	-16.54290009	-42.55099869
3527801	Lupércio	SP	0.0	0.00	-22.42749977	-49.81729889
3136603	Nova União	MG	0.0	0.00	-1.963610077	-43.57320023
3527900	Lutécia	SP	0.0	0.00	-22.32550049	-50.38570023
3136652	Juatuba	MG	0.0	0.00	-19.95700073	-44.35520172
3528007	Macatuba	SP	0.0	0.00	-22.48699951	-48.71889877
3136702	Juiz de Fora	MG	1.0	0.00	-21.74640083	-43.46569824
3202256	Governador Lindenberg	ES	0.0	0.00	-19.20639992	-40.50040054
3528106	Macaubal	SP	0.0	0.00	-20.83810043	-49.97529984
3302858	Mesquita	RJ	0.0	0.00	-22.80120087	-43.44850159
3136801	Juramento	MG	0.0	0.00	-16.84070015	-43.56579971
3528205	Macedônia	SP	0.0	0.00	-20.0984993	-50.18220139
3136900	Juruaia	MG	0.0	0.00	-21.22200012	-46.52740097
3528304	Magda	SP	0.0	0.00	-20.59700012	-50.23049927
3136959	Juvenília	MG	0.0	0.00	-14.39610004	-44.09370041
3528403	Mairinque	SP	0.0	0.00	-2.350790024	-47.23009872
3137007	Ladainha	MG	25.0	1.47	-17.62689972	-41.82289886
3137106	Lagamar	MG	0.0	0.00	-18.10490036	-46.73609924
3528502	Mairiporã	SP	0.0	0.00	-23.31780052	-46.56159973
3528601	Manduri	SP	0.0	0.00	-23.04710007	-49.30400085
3137205	Lagoa da Prata	MG	0.0	0.00	-20.01169968	-4.549530029
3528700	Marabá Paulista	SP	0.0	0.00	-22.12779999	-52.05630112
3137304	Lagoa dos Patos	MG	0.0	0.00	-1.700860024	-44.64759827
3528809	Maracaí	SP	0.0	0.00	-22.65259933	-50.74620056
3137403	Lagoa Dourada	MG	0.0	0.00	-20.89279938	-44.07540131
3528858	Marapoama	SP	0.0	0.00	-21.25510025	-49.13800049
3137502	Lagoa Formosa	MG	0.0	0.00	-18.77079964	-46.33779907
3528908	Mariápolis	SP	0.0	0.00	-2.177980042	-51.17240143
3137536	Lagoa Grande	MG	0.0	0.00	-17.76350021	-46.50189972
3529005	Marília	SP	0.0	0.00	-22.17849922	-49.98329926
3137601	Lagoa Santa	MG	0.0	0.00	-19.62849998	-43.88759995
3529104	Marinópolis	SP	0.0	0.00	-20.48069954	-5.083269882
3137700	Lajinha	MG	8.0	0.41	-20.14410019	-41.58929825
3529203	Martinópolis	SP	0.0	0.00	-22.17259979	-51.12369919
3137809	Lambari	MG	0.0	0.00	-22.00180054	-45.3783989
3529302	Matão	SP	0.0	0.00	-21.61039925	-4.843109894
3137908	Lamim	MG	0.0	0.00	-20.78059959	-43.47539902
3529401	Mauá	SP	0.0	0.00	-23.66589928	-46.44729996
3138005	Laranjal	MG	0.0	0.00	-21.35740089	-42.45199966
3529500	Mendonça	SP	0.0	0.00	-21.19379997	-49.57049942
3138104	Lassance	MG	0.0	0.00	-17.88190079	-44.69620132
3529609	Meridiano	SP	0.0	0.00	-20.3987999	-50.18730164
3138203	Lavras	MG	0.0	0.00	-21.26589966	-45.03969955
3529658	Mesópolis	SP	0.0	0.00	-19.95779991	-50.61529922
3138302	Leandro Ferreira	MG	0.0	0.00	-19.67989922	-45.04190063
3529708	Miguelópolis	SP	0.0	0.00	-20.18569946	-48.10400009
3138351	Leme do Prado	MG	0.0	0.00	-17.07089996	-42.74489975
3529807	Mineiros do Tietê	SP	0.0	0.00	-22.46319962	-48.43370056
3138401	Leopoldina	MG	1.0	1.96	-21.5496006	-42.64490128
3529906	Miracatu	SP	0.0	0.00	-24.19519997	-47.39500046
3138500	Liberdade	MG	0.0	0.00	-22.01670074	-44.33399963
3530003	Mira Estrela	SP	0.0	0.00	-19.95249939	-50.12459946
3138609	Lima Duarte	MG	0.0	0.00	-21.8003006	-43.88790131
3530102	Mirandópolis	SP	0.0	0.00	-21.08139992	-5.113140106
3138625	Limeira do Oeste	MG	0.0	0.00	-19.40780067	-50.64849854
3530201	Mirante do Paranapanema	SP	0.0	0.00	-22.34980011	-5.199829865
3138658	Lontra	MG	0.0	0.00	-1.585700035	-44.27009964
3530300	Mirassol	SP	0.0	0.00	-2.082799912	-49.50479889
3138674	Luisburgo	MG	0.0	0.00	-20.43709946	-42.07540131
3530409	Mirassolândia	SP	0.0	0.00	-2.059449959	-49.48740005
3138682	Luislândia	MG	0.0	0.00	-16.19470024	-44.59659958
3530508	Mococa	SP	0.0	0.00	-21.44980049	-47.03310013
3138708	Luminárias	MG	0.0	0.00	-21.53899956	-44.92399979
3530607	Moji das Cruzes	SP	0.0	0.00	-23.56959915	-46.18659973
3138807	Luz	MG	0.0	0.00	-1.982799912	-45.68030167
3530706	Mogi Guaçu	SP	0.0	0.00	-2.223200035	-47.02959824
3138906	Machacalis	MG	0.0	0.00	-17.08550072	-40.72060013
3530805	Moji-Mirim	SP	0.0	0.00	-22.4510994	-4.698889923
3114709	Carvalhópolis	MG	0.0	0.00	-21.77400017	-4.583119965
3530904	Mombuca	SP	0.0	0.00	-2.29428997	-47.6026001

3139102	Madre de Deus de Minas	MG	0.0	0.00	-2.148600006	-44.33330154
3531001	Monções	SP	0.0	0.00	-20.86370087	-50.07989883
3139201	Malacacheta	MG	11.0	5.86	-17.84469986	-42.11190033
3531100	Mongaguá	SP	0.0	0.00	-24.07060051	-46.66820145
3139250	Mamonas	MG	0.0	0.00	-15.01640034	-42.95560074
3531209	Monte Alegre do Sul	SP	0.0	0.00	-0.227047005	-46.66799927
3139300	Manga	MG	0.0	0.00	-14.64859962	-44.09389877
3531308	Monte Alto	SP	0.0	0.00	-21.25919914	-48.53350067
3139409	Manhuaçu	MG	5.0	6.28	-20.19580078	-42.10770035
3531407	Monte Aprazível	SP	0.0	0.00	-20.73609924	-49.7682991
3139508	Manhumirim	MG	1.0	4.68	-20.34830093	-41.94029999
3531506	Monte Azul Paulista	SP	0.0	0.00	-20.90920067	-48.68180084
3139607	Mantena	MG	0.0	0.00	-18.68589973	-4.108119965
3531605	Monte Castelo	SP	0.0	0.00	-21.25530052	-51.5719986
3139706	Maravilhas	MG	0.0	0.00	-19.50169945	-4.467200089
3531704	Monteiro Lobato	SP	0.0	0.00	-22.93689919	-45.80479813
3139805	Mar de Espanha	MG	0.0	0.00	-21.87849998	-43.01979828
3531803	Monte Mor	SP	0.0	0.00	-22.95350075	-4.730780029
3139904	Maria da Fé	MG	0.0	0.00	-22.3234005	-45.31430054
3531902	Morro Agudo	SP	0.0	0.00	-2.068899918	-48.15390015
3140001	Mariana	MG	1.0	1.84	-20.32990074	-43.33240128
3532009	Morungaba	SP	0.0	0.00	-22.89389992	-46.78359985
3140100	Marilac	MG	0.0	0.00	-18.49410057	-42.07600021
3532058	Motuca	SP	0.0	0.00	-2.151399994	-48.17070007
3140159	Mário Campos	MG	0.0	0.00	-20.07719994	-4.417639923
3532108	Murutinga do Sul	SP	0.0	0.00	-20.99720001	-5.130170059
3140209	Maripá de Minas	MG	0.0	0.00	-2.169540024	-42.95759964
3532157	Nantes	SP	0.0	0.00	-22.59729958	-51.20130157
3140308	Marliéria	MG	0.0	0.00	-19.70630074	-42.61529922
3532207	Narandiba	SP	0.0	0.00	-22.56430054	-51.52209854
3140407	Marmelópolis	MG	0.0	0.00	-22.46430016	-45.17359924
3532306	Natividade da Serra	SP	0.0	0.00	-23.41530037	-45.38199997
3140506	Martinho Campos	MG	0.0	0.00	-1.941410065	-45.19139862
3532405	Nazaré Paulista	SP	0.0	0.00	-23.19169998	-46.3667984
3140530	Martins Soares	MG	0.0	0.00	-20.26000023	-41.84370041
3532504	Neves Paulista	SP	0.0	0.00	-20.86779976	-49.64770126
3140555	Mata Verde	MG	0.0	0.00	-15.76229954	-40.7030983
3532603	Nhandeara	SP	0.0	0.00	-20.67329979	-50.04729843
3140605	Materlândia	MG	0.0	0.00	-18.46220016	-43.04800034
3532702	Nipoã	SP	0.0	0.00	-20.8920002	-4.977830124
3140704	Mateus Leme	MG	0.0	0.00	-20.02840042	-44.43799973
3532801	Nova Aliança	SP	0.0	0.00	-2.10625E-05	-49.51649857
3140803	Matias Barbosa	MG	0.0	0.00	-2.187179947	-43.3091011
3532827	Nova Campina	SP	0.0	0.00	-24.1909008	-48.97320175
3140852	Matias Cardoso	MG	0.0	0.00	-14.90330029	-4.376229858
3532843	Nova Canaã Paulista	SP	0.0	0.00	-20.37220001	-50.91080093
3140902	Matipó	MG	2.0	1.13	-20.31220055	-42.31919861
3532868	Nova Castilho	SP	0.0	0.00	-20.77799988	-50.3465004
3141009	Mato Verde	MG	0.0	0.00	-15.42599964	-42.86439896
3532900	Nova Europa	SP	0.0	0.00	-21.77429962	-48.55009842
3141108	Matozinhos	MG	0.0	0.00	-19.52479935	-44.05239868
3533007	Nova Granada	SP	0.0	0.00	-20.47640038	-49.33169937
3141207	Matutina	MG	0.0	0.00	-19.19440079	-45.99729919
3533106	Nova Guataporanga	SP	0.0	0.00	-21.32279968	-51.64590073
3141306	Medeiros	MG	0.0	0.00	-19.99069977	-4.634080124
3533205	Nova Independência	SP	0.0	0.00	-21.13570023	-51.50569916
3141405	Medina	MG	0.0	0.00	-16.27140045	-41.51129913
3533254	Novais	SP	0.0	0.00	-20.98870087	-48.91839981
3141504	Mendes Pimentel	MG	0.0	0.00	-1.86303997	-41.35549927
3533304	Nova Luzitânia	SP	0.0	0.00	-20.8689003	-50.24990082
3141603	Mercês	MG	0.0	0.00	-21.18989944	-43.33530045
3533403	Nova Odessa	SP	0.0	0.00	-22.78440094	-47.28519821
3141702	Mesquita	MG	0.0	0.00	-19.24810028	-42.61299896
3533502	Novo Horizonte	SP	0.0	0.00	-21.46929932	-49.28430176
3141801	Minas Novas	MG	4.0	1.30	-17.36230087	-42.43610001
3533601	Nuporanga	SP	0.0	0.00	-20.70479965	-47.72949982
3141900	Minduri	MG	0.0	0.00	-2.167569923	-44.6147995
3533700	Ocauçu	SP	0.0	0.00	-22.43849945	-49.93840027
3142007	Mirabela	MG	0.0	0.00	-16.26049995	-4.415800095
3533809	Óleo	SP	0.0	0.00	-22.95389938	-49.38339996
3142106	Miradouro	MG	0.0	0.00	-20.85000038	-42.40060043
3533908	Olímpia	SP	0.0	0.00	-2.070549965	-48.95970154
3142205	Miraí	MG	0.0	0.00	-21.15139961	-42.6189003
3534005	Onda Verde	SP	0.0	0.00	-20.61790085	-49.2439003
3142254	Miravânia	MG	0.0	0.00	-14.75360012	-44.42300034
3534104	Oriente	SP	0.0	0.00	-22.14229965	-50.09389877
3142304	Moeda	MG	0.0	0.00	-2.033119965	-43.99340057
3534203	Orindiúva	SP	0.0	0.00	-20.20240021	-49.35649872
3142403	Moema	MG	0.0	0.00	-19.8423996	-45.40879822
3534302	Orlândia	SP	0.0	0.00	-20.69890022	-47.90660095
3142502	Monjolos	MG	0.0	0.00	-18.3885994	-44.02399826
3534401	Osasco	SP	0.0	0.00	-23.5291996	-46.78950119
3142601	Monsenhor Paulo	MG	0.0	0.00	-21.73399925	-45.48310089
3534500	Oscar Bressane	SP	0.0	0.00	-22.29520035	-5.025759888
3142700	Montalvânia	MG	0.0	0.00	-1.446730042	-44.50099945
3534609	Osvaldo Cruz	SP	0.0	0.00	-21.73320007	-50.87419891
3142809	Monte Alegre de Minas	MG	0.0	0.00	-18.84530067	-48.88710022

3534708	Ourinhos	SP	0.0	0.00	-22.95319939	-49.85279846
3142908	Monte Azul	MG	0.0	0.00	-15.20790005	-42.96910095
3534757	Ouroeste	SP	0.0	0.00	-19.92340088	-50.40200043
3143005	Monte Belo	MG	0.0	0.00	-21.31559944	-46.32740021
3534807	Ouro Verde	SP	0.0	0.00	-21.51059914	-51.73799896
3143104	Monte Carmelo	MG	0.0	0.00	-18.7105999	-47.46160126
3534906	Pacaembu	SP	0.0	0.00	-21.49559975	-51.2745018
3143153	Monte Formoso	MG	0.0	0.00	-16.87940025	-41.26649857
3535002	Palestina	SP	0.0	0.00	-20.32159996	-49.49860001
3143203	Monte Santo de Minas	MG	0.0	0.00	-21.18959999	-46.94749832
3535101	Palmares Paulista	SP	0.0	0.00	-21.10449982	-48.82699966
3143302	Montes Claros	MG	1.0	0.00	-16.62150002	-43.93119812
3535200	Palmeira d'Oeste	SP	0.0	0.00	-20.43910027	-50.7492981
3143401	Monte Sião	MG	0.0	0.00	-22.42090034	-46.51979828
3535309	Palmital	SP	0.0	0.00	-2.282900047	-50.22109985
3143450	Montezuma	MG	0.0	0.00	-15.19180012	-42.46979904
3535408	Panorama	SP	0.0	0.00	-21.46310043	-51.85760117
3143500	Morada Nova de Minas	MG	0.0	0.00	-18.57929993	-45.4034996
3535507	Paraguaçu Paulista	SP	0.0	0.00	-22.45910072	-50.6269989
3143609	Morro da Garça	MG	0.0	0.00	-18.63089943	-44.63410187
3535606	Paraibuna	SP	0.0	0.00	-23.47830009	-45.64210129
3143708	Morro do Pilar	MG	0.0	0.00	-19.23609924	-43.40390015
3535705	Paraíso	SP	0.0	0.00	-21.0196991	-48.76520157
3143807	Munhoz	MG	0.0	0.00	-22.63260078	-46.30540085
3535804	Paranapanema	SP	0.0	0.00	-23.43840027	-48.79579926
3143906	Muriaé	MG	0.0	0.00	-21.09189987	-42.42250061
3535903	Paranapuã	SP	0.0	0.00	-20.06450081	-50.5945015
3144003	Mutum	MG	1.0	3.75	-19.92000008	-41.44789886
3536000	Parapuã	SP	0.0	0.00	-21.85320091	-50.82180023
3144102	Muzambinho	MG	0.0	0.00	-2.135630035	-0.465172005
3536109	Pardinho	SP	0.0	0.00	-2.310239983	-48.40280151
3144201	Nacip Raydan	MG	0.0	0.00	-18.48060036	-42.18999863
3536208	Pariquera-Açu	SP	0.0	0.00	-24.67869949	-47.8504982
3144300	Nanuque	MG	1.0	2.45	-17.7798996	-40.49710083
3536257	Parisi	SP	0.0	0.00	-20.27129936	-50.03789902
3144359	Naque	MG	0.0	0.00	-19.18169975	-42.32490158
3536307	Patrocínio Paulista	SP	0.0	0.00	-20.70499992	-47.29029846
3144375	Natalândia	MG	0.0	0.00	-16.54560089	-46.4756012
3536406	Paulicéia	SP	0.0	0.00	-21.19890022	-51.79109955
3144409	Natércia	MG	0.0	0.00	-22.13080025	-45.51210022
3536505	Paulínia	SP	0.0	0.00	-22.74819946	-47.14530182
3144508	Nazareno	MG	0.0	0.00	-21.20479965	-44.61930084
3536570	Paulistânia	SP	0.0	0.00	-22.5685997	-49.30630112
3144607	Nepomuceno	MG	0.0	0.00	-21.22890091	-45.26179886
3536604	Paulo de Faria	SP	0.0	0.00	-20.06690025	-49.46419907
3144656	Ninheira	MG	0.0	0.00	-15.3682003	-41.66009903
3536703	Pederneiras	SP	0.0	0.00	-22.30120087	-48.86159897
3144672	Nova Belém	MG	0.0	0.00	-1.848920059	-41.10250092
3536802	Pedra Bela	SP	0.0	0.00	-22.77569962	-46.44210052
3144706	Nova Era	MG	0.0	0.00	-19.71990013	-43.01440048
3536901	Pedranópolis	SP	0.0	0.00	-20.21039963	-50.1072998
3144805	Nova Lima	MG	0.0	0.00	-20.07480049	-4.390480042
3537008	Pedregulho	SP	0.0	0.00	-20.20750046	-47.43909836
3144904	Nova Módica	MG	0.0	0.00	-18.45319939	-41.52280045
3145000	Nova Ponte	MG	0.0	0.00	-19.26199913	-47.70740128
3537107	Pedreira	SP	0.0	0.00	-22.75460052	-46.8891983
3537156	Pedrinhas Paulista	SP	0.0	0.00	-22.81710052	-50.80329895
3145059	Nova Porteirinha	MG	0.0	0.00	-15.73589993	-43.27560043
3537206	Pedro de Toledo	SP	0.0	0.00	-24.16379929	-47.16379929
3145109	Nova Resende	MG	0.0	0.00	-21.0984993	-46.41650009
3537305	Penápolis	SP	0.0	0.00	-21.40530014	-50.09629822
3145208	Nova Serrana	MG	0.0	0.00	-19.85289955	-44.97480011
3537404	Pereira Barreto	SP	0.0	0.00	-20.67819977	-51.10680008
3145307	Novo Cruzeiro	MG	49.0	1.59	-17.37129974	-41.95569992
3537503	Pereiras	SP	0.0	0.00	-23.12100029	-47.97940063
3145356	Novo Oriente de Minas	MG	2.0	1.93	-17.23060036	-41.22800064
3145372	Novorizonte	MG	0.0	0.00	-16.01059914	-42.40209961
3537701	Piacatu	SP	0.0	0.00	-21.57130051	-50.64400101
3145406	Olaria	MG	0.0	0.00	-21.90810013	-43.96720123
3537800	Piedade	SP	0.0	0.00	-23.78720093	-47.43870163
3145455	Olhos-d'Água	MG	0.0	0.00	-17.47900009	-4.359550095
3537909	Pilar do Sul	SP	0.0	0.00	-23.8567009	-47.72890091
3145505	Olímpio Noronha	MG	0.0	0.00	-22.09110069	-4.528590012
3538006	Pindamonhangaba	SP	0.0	0.00	-22.88010025	-4.545940018
3145604	Oliveira	MG	0.0	0.00	-20.7507	-44.73709869
3538105	Pindorama	SP	0.0	0.00	-21.20980072	-48.91189957
3145703	Oliveira Fortes	MG	0.0	0.00	-21.33690071	-43.51580048
3538204	Pinhalzinho	SP	0.0	0.00	-22.77910042	-46.57369995
3145802	Onça de Pitangui	MG	0.0	0.00	-19.70899963	-44.7364006
3538303	Piquerobi	SP	0.0	0.00	-21.85339928	-51.73210144
3145851	Oratórios	MG	0.0	0.00	-2.042670059	-42.79499817
3538501	Piquete	SP	0.0	0.00	-22.59049988	-45.17399979
3145877	Orizânia	MG	0.0	0.00	-20.51490021	-42.21369934
3538600	Piracaia	SP	0.0	0.00	-23.04719925	-46.30339813
3145901	Ouro Branco	MG	0.0	0.00	-20.52630043	-43.6753006
3538709	Piracicaba	SP	0.0	0.00	-22.72640038	-47.78310013
3146008	Ouro Fino	MG	0.0	0.00	-22.25569916	-46.38470078

3538808	Piraju	SP	0.0	0.00	-23.18700027	-49.36650085
3146107	Ouro Preto	MG	0.0	0.00	-20.39179993	-43.61119843
3538907	Pirajuí	SP	0.0	0.00	-21.95739937	-4.941389847
3146206	Ouro Verde de Minas	MG	1.0	1.66	-18.04879951	-41.29660034
3539004	Pirangi	SP	0.0	0.00	-21.09390068	-48.6697998
3146255	Padre Carvalho	MG	0.0	0.00	-16.30200005	-42.58639908
3539103	Pirapora do Bom Jesus	SP	0.0	0.00	-23.3784008	-46.98509979
3146305	Padre Paraíso	MG	1.0	0.53	-17.03930092	-41.55250168
3539202	Pirapozinho	SP	0.0	0.00	-22.47990036	-51.62049866
3146404	Paineiras	MG	0.0	0.00	-18.92009926	-45.49229813
3539301	Pirassununga	SP	0.0	0.00	-2.199609947	-4.739049912
3146503	Pains	MG	0.0	0.00	-20.38229942	-45.69599915
3539400	Piratininga	SP	0.0	0.00	-22.4260006	-49.19260025
3146552	Pai Pedro	MG	0.0	0.00	-1.542080021	-43.1427002
3539509	Pitangueiras	SP	0.0	0.00	-21.00639915	-48.24990082
3146602	Paiva	MG	0.0	0.00	-21.28800011	-43.42470169
3539608	Planalto	SP	0.0	0.00	-21.00139999	-49.93629837
3146701	Palma	MG	0.0	0.00	-21.42009926	-42.33010101
3539707	Platina	SP	0.0	0.00	-22.6257	-50.21319962
3146750	Palmópolis	MG	0.0	0.00	-16.7887001	-40.37210083
3539806	Poá	SP	0.0	0.00	-23.53019905	-4.634669876
3146909	Papagaios	MG	0.0	0.00	-19.37730026	-44.69179916
3539905	Poloni	SP	0.0	0.00	-20.75259972	-49.8205986
3147006	Paracatu	MG	0.0	0.00	-17.1746006	-4.688460159
3540002	Pompéia	SP	0.0	0.00	-22.02939987	-50.18769836
3147105	Pará de Minas	MG	0.0	0.00	-19.83519936	-44.60860062
3540101	Pongaí	SP	0.0	0.00	-21.7303009	-49.35900116
3147204	Paraguaçu	MG	0.0	0.00	-2.15678997	-45.75080109
3540200	Pontal	SP	0.0	0.00	-20.98489952	-48.06529999
3147303	Paraisópolis	MG	0.0	0.00	-2.257259941	-45.81800079
3540259	Pontalinda	SP	0.0	0.00	-20.45100021	-50.52539825
3147402	Paraopeba	MG	0.0	0.00	-19.2730999	-44.45389938
3540309	Pontes Gestal	SP	0.0	0.00	-20.17539978	-49.7580986
3147501	Passabém	MG	0.0	0.00	-1.936100006	-43.17630005
3540408	Populina	SP	0.0	0.00	-19.90780067	-50.51689911
3147600	Passa Quatro	MG	0.0	0.00	-22.40649986	-44.9693985
3540507	Porangaba	SP	0.0	0.00	-23.17399979	-48.11790085
3147709	Passa Tempo	MG	0.0	0.00	-20.64979935	-44.49229813
3540606	Porto Feliz	SP	0.0	0.00	-23.23049927	-4.751699829
3147808	Passa-Vinte	MG	0.0	0.00	-22.18379974	-44.26259995
3540705	Porto Ferreira	SP	1.0	1.95	-21.84189987	-47.4469986
3147907	Passos	MG	0.0	0.00	-2.072019959	-46.62250137
3540754	Potim	SP	0.0	0.00	-22.82550049	-45.30360031
3147956	Patis	MG	0.0	0.00	-16.0814991	-44.10229874
3540804	Potirendaba	SP	0.0	0.00	-21.0753994	-4.939199829
3148004	Patos de Minas	MG	0.0	0.00	-18.59810066	-46.50889969
3540853	Pracinha	SP	0.0	0.00	-21.83779907	-51.07540131
3148103	Patrocínio	MG	1.0	1.21	-18.9715004	-47.05160141
3540903	Pradópolis	SP	0.0	0.00	-21.34480095	-48.0862999
3148202	Patrocínio do Muriaé	MG	0.0	0.00	-21.1685009	-42.2533989
3541000	Praia Grande	SP	0.0	0.00	-24.01580048	-46.52199936
3148301	Paula Cândido	MG	0.0	0.00	-20.85650063	-4.298600006
3541059	Pratânia	SP	0.0	0.00	-22.81830025	-48.69940186
3148400	Paulistas	MG	0.0	0.00	-18.45849991	-42.86619949
3541109	Presidente Alves	SP	0.0	0.00	-22.12170029	-49.43220139
3148509	Pavão	MG	0.0	0.00	-17.47260094	-41.06280136
3541208	Presidente Bernardes	SP	0.0	0.00	-22.10820007	-51.62039948
3148608	Peçanha	MG	0.0	0.00	-18.55739975	-42.51129913
3541307	Presidente Epitácio	SP	0.0	0.00	-21.9102993	-52.17359924
3148707	Pedra Azul	MG	0.0	0.00	-15.95440006	-41.18730164
3541406	Presidente Prudente	SP	0.0	0.00	-21.9932003	-51.34329987
3148756	Pedra Bonita	MG	0.0	0.00	-20.48159981	-42.37540054
3541505	Presidente Venceslau	SP	0.0	0.00	-21.79330063	-51.83850098
3148806	Pedra do Anta	MG	0.0	0.00	-2.059620094	-42.71879959
3541604	Promissão	SP	0.0	0.00	-21.52519989	-49.87099838
3148905	Pedra do Indaiá	MG	0.0	0.00	-20.28790092	-45.2303009
3541653	Quadra	SP	0.0	0.00	-23.30089951	-48.03919983
3149002	Pedra Dourada	MG	0.0	0.00	-20.82830048	-42.1556015
3541703	Quatá	SP	0.0	0.00	-22.20669937	-50.64569855
3149101	Pedralva	MG	0.0	0.00	-22.25060081	-0.454547005
3541802	Queiroz	SP	0.0	0.00	-21.80060005	-50.24489975
3149150	Pedras de Maria da Cruz	MG	0.0	0.00	-15.62660027	-4.432479858
3149200	Pedrinópolis	MG	0.0	0.00	-19.19309998	-47.51119995
3541901	Queluz	SP	0.0	0.00	-22.50440025	-44.78559875
3542008	Quintana	SP	0.0	0.00	-2.209989929	-50.36930084
3149309	Pedro Leopoldo	MG	0.0	0.00	-19.6420002	-44.0542984
3542107	Rafard	SP	0.0	0.00	-23.04579926	-47.58940125
3149408	Pedro Teixeira	MG	0.0	0.00	-21.72649956	-43.73020172
3542206	Rancharia	SP	0.0	0.00	-22.28289986	-50.92860031
3149507	Pequeri	MG	0.0	0.00	-21.82679939	-43.13299942
3542305	Redenção da Serra	SP	0.0	0.00	-23.25370026	-45.52669907
3149606	Pequi	MG	0.0	0.00	-19.60549927	-44.63650131
3542404	Regente Feijó	SP	0.0	0.00	-22.25090027	-51.29449844
3149705	Perdigão	MG	0.0	0.00	-19.9409008	-45.05889893
3542503	Reginópolis	SP	0.0	0.00	-21.88339996	-49.18080139
3149804	Perdizes	MG	0.0	0.00	-1.940080071	-47.19630051
3542602	Registro	SP	0.0	0.00	-24.50379944	-47.81969833

3149903	Perdões	MG	0.0	0.00	-2.107900047	-45.06650162
3542701	Restinga	SP	0.0	0.00	-20.64819908	-47.50650024
3149952	Periquito	MG	0.0	0.00	-19.08429909	-42.2322998
3542800	Ribeira	SP	0.0	0.00	-24.60810089	-49.03720093
3150000	Pescador	MG	1.0	2.42	-18.33180046	-41.56090164
3542909	Ribeirão Bonito	SP	0.0	0.00	-22.05990028	-48.18590164
3150109	Piau	MG	0.0	0.00	-21.50309944	-43.31779861
3543006	Ribeirão Branco	SP	0.0	0.00	-24.25639915	-48.7784996
3150158	Piedade de Caratinga	MG	7.0	0.98	-1.976329994	-0.420452995
3543105	Ribeirão Corrente	SP	0.0	0.00	-20.44879913	-47.57350159
3150208	Piedade de Ponte Nova	MG	0.0	0.00	-20.24239922	-42.71929932
3543204	Ribeirão do Sul	SP	0.0	0.00	-22.75180054	-49.92300034
3150307	Piedade do Rio Grande	MG	0.0	0.00	-21.48509979	-44.15670013
3543238	Ribeirão dos Índios	SP	0.0	0.00	-21.78470039	-51.58380127
3150406	Piedade dos Gerais	MG	0.0	0.00	-20.47470093	-44.24720001
3543253	Ribeirão Grande	SP	0.0	0.00	-24.18939972	-48.35910034
3150505	Pimenta	MG	0.0	0.00	-20.53129959	-45.83229828
3543303	Ribeirão Pires	SP	0.0	0.00	-23.70129967	-46.40340042
3150539	Pingo-d'Água	MG	0.0	0.00	-19.74440002	-42.42229843
3543402	Ribeirão Preto	SP	0.0	0.00	-21.21120071	-47.82149887
3150570	Pintópolis	MG	0.0	0.00	-16.05109978	-45.24840164
3543501	Riversul	SP	0.0	0.00	-23.84720039	-49.44820023
3150604	Piracema	MG	0.0	0.00	-20.52319908	-44.42369843
3150703	Pirajuba	MG	0.0	0.00	-19.9409008	-48.65840149
3543600	Rifaina	SP	0.0	0.00	-20.06209946	-47.44200134
3543709	Rincão	SP	0.0	0.00	-21.59390068	-48.03179932
3150802	Piranga	MG	0.0	0.00	-20.63430023	-43.29980087
3543808	Rinópolis	SP	0.0	0.00	-21.68120003	-50.71780014
3150901	Piranguçu	MG	0.0	0.00	-22.55669975	-45.51470184
3543907	Rio Claro	SP	0.0	0.00	-22.37360001	-47.58000183
3151008	Piranguinho	MG	0.0	0.00	-2.236389923	-4.558909988
3544004	Rio das Pedras	SP	0.0	0.00	-0.228509007	-47.59930038
3151107	Pirapetinga	MG	0.0	0.00	-21.67980003	-42.36439896
3544103	Rio Grande da Serra	SP	0.0	0.00	-23.73839951	-46.38000107
3151206	Pirapora	MG	0.0	0.00	-1.740010071	-44.86790085
3544202	Riolândia	SP	0.0	0.00	-20.02420044	-49.70999908
3151305	Piraúba	MG	0.0	0.00	-21.26499939	-43.0306015
3544251	Rosana	SP	0.0	0.00	-2.248920059	-52.83720016
3151404	Pitangui	MG	0.0	0.00	-19.58849907	-44.8871994
3151503	Piumhi	MG	1.0	3.14	-20.44239998	-46.05130005
3544301	Roseira	SP	0.0	0.00	-22.93330002	-45.30260086
3544400	Rubiácea	SP	0.0	0.00	-21.36129951	-50.78639984
3151602	Planura	MG	0.0	0.00	-20.08160019	-48.64670181
3544509	Rubinéia	SP	0.0	0.00	-20.24810028	-51.01810074
3151701	Poço Fundo	MG	0.0	0.00	-21.80690002	-45.99440002
3544608	Sabino	SP	0.0	0.00	-21.46929932	-49.57839966
3151800	Poços de Caldas	MG	0.0	0.00	-21.80850029	-46.56380081
3544707	Sagres	SP	0.0	0.00	-21.86770058	-5.099039841
3151909	Pocrane	MG	1.0	1.11	-1.958589935	-41.55810165
3544806	Sales	SP	0.0	0.00	-21.34720039	-49.50310135
3152006	Pompéu	MG	0.0	0.00	-19.13190079	-44.92480087
3544905	Sales Oliveira	SP	0.0	0.00	-20.83230019	-4.785480118
3152105	Ponte Nova	MG	0.0	0.00	-20.41300011	-4.291859818
3545001	Salesópolis	SP	0.0	0.00	-23.5802002	-45.84320068
3152131	Ponto Chique	MG	0.0	0.00	-16.60919952	-44.97480011
3545100	Salmourão	SP	0.0	0.00	-21.58869934	-50.87689972
3152170	Ponto dos Volantes	MG	0.0	0.00	-16.83550072	-4.147489929
3545159	Saltinho	SP	0.0	0.00	-22.87140083	-47.73329926
3152204	Porteirinha	MG	0.0	0.00	-15.70880032	-43.08250046
3545209	Salto	SP	0.0	0.00	-23.18029976	-47.30279922
3152303	Porto Firme	MG	0.0	0.00	-20.66449928	-43.08169937
3545308	Salto de Pirapora	SP	0.0	0.00	-23.6529007	-47.58069992
3152402	Poté	MG	31.0	1.98	-17.81509972	-41.77270126
3545407	Salto Grande	SP	0.0	0.00	-22.8708992	-49.96160126
3152501	Pouso Alegre	MG	0.0	0.00	-22.26600075	-45.94079971
3545506	Sandovalina	SP	0.0	0.00	-22.47800064	-51.84209824
3152600	Pouso Alto	MG	0.0	0.00	-22.17620087	-44.93349838
3545605	Santa Adélia	SP	0.0	0.00	-21.31809998	-48.81930161
3152709	Prados	MG	0.0	0.00	-21.09959984	-44.0644989
3545704	Santa Albertina	SP	0.0	0.00	-20.01460075	-50.72790146
3152808	Prata	MG	0.0	0.00	-19.33060074	-48.95460129
3545803	Santa Bárbara d'Oeste	SP	0.0	0.00	-22.80080032	-47.42850113
3152907	Pratápolis	MG	0.0	0.00	-20.78829956	-46.86320114
3153004	Pratinha	MG	0.0	0.00	-19.76210022	-46.40269852
3546009	Santa Branca	SP	0.0	0.00	-23.4260006	-4.586389923
3546108	Santa Clara d'Oeste	SP	0.0	0.00	-20.0673008	-50.90909958
3153103	Presidente Bernardes	MG	0.0	0.00	-20.77149963	-43.15579987
3546207	Santa Cruz da Conceição	SP	0.0	0.00	-22.12190056	-0.047485699
3153202	Presidente Juscelino	MG	0.0	0.00	-18.7269001	-44.08420181
3546256	Santa Cruz da Esperança	SP	0.0	0.00	-21.27479935	-47.43939972
3153301	Presidente Kubitschek	MG	0.0	0.00	-18.63439941	-0.435797005
3546306	Santa Cruz das Palmeiras	SP	0.0	0.00	-21.85580063	-47.25030136
3153400	Presidente Olegário	MG	0.0	0.00	-18.23870087	-46.33840179
3546405	Santa Cruz do Rio Pardo	SP	0.0	0.00	-22.80920029	-49.56869888
3153509	Alto Jequitibá	MG	0.0	0.00	-20.43829918	-41.94929886
3546504	Santa Ernestina	SP	0.0	0.00	-21.45240021	-48.3669014
3153608	Prudente de Morais	MG	0.0	0.00	-19.46570015	-4.411270142

3546603	Santa Fé do Sul	SP	0.0	0.00	-20.24340057	-50.95259857
3153707	Quartel Geral	MG	0.0	0.00	-19.28079987	-45.60409927
3546702	Santa Gertrudes	SP	0.0	0.00	-22.47500038	-47.52159882
3153806	Queluzito	MG	0.0	0.00	-20.7329998	-43.89160156
3546801	Santa Isabel	SP	0.0	0.00	-23.28930092	-46.24250031
3153905	Raposos	MG	0.0	0.00	-19.98220062	-43.7840004
3546900	Santa Lúcia	SP	0.0	0.00	-21.67079926	-48.05720139
3154002	Raul Soares	MG	0.0	0.00	-20.01230049	-42.39139938
3547007	Santa Maria da Serra	SP	0.0	0.00	-22.56389999	-48.15589905
3154101	Recreio	MG	0.0	0.00	-21.52099991	-42.43909836
3547106	Santa Mercedes	SP	0.0	0.00	-21.3173008	-51.73730087
3154150	Reduto	MG	0.0	0.00	-20.23870087	-41.94139862
3547205	Santana da Ponte Pensa	SP	0.0	0.00	-20.26099968	-50.79750061
3154200	Resende Costa	MG	0.0	0.00	-20.83959961	-44.29570007
3547304	Santana de Parnaíba	SP	0.0	0.00	-23.44939995	-46.91619873
3154309	Resplendor	MG	1.0	5.85	-19.21190071	-41.15119934
3547403	Santa Rita d'Oeste	SP	0.0	0.00	-20.09700012	-50.81330109
3154408	Ressaquinha	MG	0.0	0.00	-21.08670044	-43.75429916
3547502	Santa Rita do Passa Quatro	SP	0.0	0.00	-21.68239975	-47.5082016
3154457	Riachinho	MG	0.0	0.00	-16.28310013	-45.92860031
3547601	Santa Rosa de Viterbo	SP	0.0	0.00	-21.5041008	-47.36669922
3154507	Riacho dos Machados	MG	0.0	0.00	-16.05680084	-42.9939003
3547650	Santa Salete	SP	0.0	0.00	-2.025860024	-50.72029877
3154606	Ribeirão das Neves	MG	0.0	0.00	-19.78030014	-44.07339859
3547700	Santo Anastácio	SP	0.0	0.00	-0.220384007	-51.72290039
3154705	Ribeirão Vermelho	MG	0.0	0.00	-21.15600014	-0.045076801
3547809	Santo André	SP	1.0	0.00	-23.72879982	-46.4416008
3154804	Rio Acima	MG	0.0	0.00	-20.10689926	-43.77299881
3547908	Santo Antônio da Alegria	SP	0.0	0.00	-21.09259987	-47.19609833
3155009	Rio Doce	MG	0.0	0.00	-20.2159996	-42.90710068
3548005	Santo Antônio de Posse	SP	0.0	0.00	-22.60339928	-46.95000076
3155108	Rio do Prado	MG	0.0	0.00	-16.67790031	-40.55709839
3548054	Santo Antônio do Aracanguá	SP	0.0	0.00	-20.87319946	-5.055490112
3155207	Rio Espera	MG	0.0	0.00	-20.86860085	-43.49319839
3548104	Santo Antônio do Jardim	SP	0.0	0.00	-22.13059998	-46.68420029
3155306	Rio Manso	MG	0.0	0.00	-2.026799965	-44.34609985
3548203	Santo Antônio do Pinhal	SP	0.0	0.00	-22.83200073	-4.569829941
3155405	Rio Novo	MG	0.0	0.00	-2.146949959	-4.3145401
3548302	Santo Expedito	SP	0.0	0.00	-21.82530022	-51.37039948
3155504	Rio Paranaíba	MG	0.0	0.00	-19.24130058	-46.29959869
3548401	Santópolis do Aguapeí	SP	0.0	0.00	-21.66040039	-50.51850128
3155603	Rio Pardo de Minas	MG	0.0	0.00	-15.71020031	-42.55250168
3548500	Santos	SP	0.0	0.00	-23.86899948	-46.29199982
3155702	Rio Piracicaba	MG	0.0	0.00	-19.97190094	-43.15060043
3548609	São Bento do Sapucaí	SP	0.0	0.00	-22.68180084	-45.6867981
3155801	Rio Pomba	MG	0.0	0.00	-21.24939919	-43.17290115
3548708	São Bernardo do Campo	SP	0.0	0.00	-23.81340027	-46.55080032
3155900	Rio Preto	MG	0.0	0.00	-22.04179955	-43.87939835
3548807	São Caetano do Sul	SP	0.0	0.00	-23.62660027	-46.56629944
3156007	Rio Vermelho	MG	2.0	0.15	-18.25510025	-43.0564003
3548906	São Carlos	SP	0.0	0.00	-21.91900063	-47.86759949
3156106	Ritápolis	MG	0.0	0.00	-20.9864006	-44.37639999
3549003	São Francisco	SP	0.0	0.00	-20.36610031	-50.67649841
3156205	Rochedo de Minas	MG	0.0	0.00	-21.64229965	-43.02970123
3549102	São João da Boa Vista	SP	0.0	0.00	-2.197809982	-46.80239868
3156304	Rodeiro	MG	0.0	0.00	-21.21179962	-42.84209824
3549201	São João das Duas Pontes	SP	0.0	0.00	-20.40889931	-50.38380051
3156403	Romaria	MG	0.0	0.00	-1.890480042	-47.56430054
3549250	São João de Iracema	SP	0.0	0.00	-20.52129936	-50.3567009
3156452	Rosário da Limeira	MG	0.0	0.00	-20.97979927	-42.51290131
3549300	São João do Pau d'Alho	SP	0.0	0.00	-21.22380066	-51.67359924
3156502	Rubelita	MG	0.0	0.00	-16.36860085	-42.23479843
3549409	São Joaquim da Barra	SP	0.0	0.00	-2.055069923	-47.93859863
3156601	Rubim	MG	0.0	0.00	-16.4647007	-4.049509811
3549508	São José da Bela Vista	SP	0.0	0.00	-20.58749962	-47.62770081
3156700	Sabará	MG	0.0	0.00	-19.85210037	-43.77999878
3549607	São José do Barreiro	SP	0.0	0.00	-22.7493	-44.58729935
3156809	Sabinópolis	MG	0.0	0.00	-18.65180016	-43.0644989
3156908	Sacramento	MG	0.0	0.00	-19.90539932	-47.26710129
3549706	São José do Rio Pardo	SP	0.0	0.00	-21.60390091	-46.87960052
3549805	São José do Rio Preto	SP	0.0	0.00	-20.79780006	-49.3586998
3157005	Salinas	MG	0.0	0.00	-16.10989952	-42.15380096
3549904	São José dos Campos	SP	0.0	0.00	-23.09090042	-45.92900085
3157104	Salto da Divisa	MG	0.0	0.00	-16.11160088	-40.03010178
3549953	São Lourenço da Serra	SP	0.0	0.00	-23.85250092	-46.9367981
3157203	Santa Bárbara	MG	0.0	0.00	-20.03009987	-43.47629929
3550001	São Luís do Paraitinga	SP	0.0	0.00	-23.24040031	-45.25419998
3157252	Santa Bárbara do Leste	MG	9.0	1.17	-19.94529915	-42.10979843
3550100	São Manuel	SP	0.0	0.00	-22.68230057	-48.54100037
3157278	Santa Bárbara do Monte Verde	MG	0.0	0.00	-21.96450043	-43.6957016
3550209	São Miguel Arcanjo	SP	0.0	0.00	-23.9144001	-47.99409866
3157302	Santa Bárbara do Tugúrio	MG	0.0	0.00	-21.24340057	-43.52410126
3550308	São Paulo	SP	7.0	0.00	-23.65089989	-46.64849854
3157336	Santa Cruz de Minas	MG	0.0	0.00	-21.12210083	-44.2159996
3550407	São Pedro	SP	0.0	0.00	-2.255929947	-4.792520142
3157377	Santa Cruz de Salinas	MG	0.0	0.00	-16.0515995	-41.79069901
3550506	São Pedro do Turvo	SP	0.0	0.00	-22.67939949	-49.77249908

3157401	Santa Cruz do Escalvado	MG	0.0	0.00	-20.23119926	-42.81499863
3550605	São Roque	SP	0.0	0.00	-2.354920006	-47.11029816
3157500	Santa Efigênia de Minas	MG	0.0	0.00	-18.86300087	-42.40499878
3202405	Guarapari	ES	2.0	1.90	-20.58550072	-4.054700089
3205200	Vila Velha	ES	4.0	0.00	-20.43499947	-4.037969971
3300100	Angra dos Reis	RJ	0.0	0.00	-22.98649979	-44.35319901
3302007	Itaguaí	RJ	0.0	0.00	-2.284519959	-43.81729889
3302403	Macaé	RJ	0.0	0.00	-22.2954998	-41.97539902
3302601	Mangaratiba	RJ	0.0	0.00	-22.95420074	-44.04460144
3303807	Parati	RJ	0.0	0.00	-23.14920044	-4.470640183
3304557	Rio de Janeiro	RJ	2.0	0.00	-22.92429924	-43.45259857
3304904	São Gonçalo	RJ	0.0	0.00	-22.82600021	-42.99739838
3506359	Bertioga	SP	0.0	0.00	-23.7602005	-46.03039932
3510500	Caraguatatuba	SP	0.0	0.00	-23.63969994	-45.48849869
3520400	Ilhabela	SP	0.0	0.00	-23.84880066	-45.32429886
3537602	Peruíbe	SP	0.0	0.00	-24.2791996	-47.01259995
3550704	São Sebastião	SP	0.0	0.00	-23.75309944	-45.6072998
3555406	Ubatuba	SP	1.0	1.27	-23.38190079	-45.02470016
3205309	Vitória	ES	5.0	1.53	-2.027980042	-40.29899979
